# Silicon Nanostructures for Hydrogen Generation and Storage

**DOI:** 10.3390/nano15191531

**Published:** 2025-10-07

**Authors:** Gauhar Mussabek, Gulmira Yar-Mukhamedova, Sagi Orazbayev, Valeriy Skryshevsky, Vladimir Lysenko

**Affiliations:** 1 National Nanotechnology Laboratory of Open Type, Al-Farabi Kazakh National University, Almaty 050040, Kazakhstan; gulmira.yar-muhamedova@kaznu.edu.kz (G.Y.-M.); sagi.orazbayev@kaznu.edu.kz (S.O.); 2 Institute of Information and Computational Technologies, Almaty 050072, Kazakhstan; 3 Educational Scientific Institute of High Technologies, Taras Shevchenko National University of Kyiv, 01601 Kyiv, Ukraine; skryshevsky@knu.ua; 4 Corporation Science Park, Taras Shevchenko University of Kyiv, 01033 Kyiv, Ukraine; 5 Light Matter Institute, UMR-5306, Claude Bernard University of Lyon/CNRS, Université de Lyon, 69622 Villeurbanne Cedex, France; vladimir.lysenko@univ-lyon1.fr

**Keywords:** silicon nanostructures, hydrogen generation, hydrogen storage, porous silicon, silicon composites

## Abstract

Today, hydrogen is already widely regarded as up-and-coming source of energy. It is essential to meet energy needs while reducing environmental pollution, since it has a high energy capacity and does not emit carbon oxide when burned. However, for the widespread application of hydrogen energy, it is necessary to search new technical solutions for both its production and storage. A promising effective and cost-efficient method of hydrogen generation and storage can be the use of solid materials, including nanomaterials in which chemical or physical adsorption of hydrogen occurs. Focusing on the recommendations of the DOE, the search is underway for materials with high gravimetric capacity more than 6.5% wt% and in which sorption and release of hydrogen occurs at temperatures from −20 to +100 °C and normal pressure. This review aims to summarize research on hydrogen generation and storage using silicon nanostructures and silicon composites. Hydrogen generation has been observed in Si nanoparticles, porous Si, and Si nanowires. Regardless of their size and surface chemistry, the silicon nanocrystals interact with water/alcohol solutions, resulting in their complete oxidation, the hydrolysis of water, and the generation of hydrogen. In addition, porous Si nanostructures exhibit a large internal specific surface area covered by SiH_x_ bonds. A key advantage of porous Si nanostructures is their ability to release molecular hydrogen through the thermal decomposition of SiHx groups or in interaction with water/alkali. The review also covers simulations and theoretical modeling of H_2_ generation and storage in silicon nanostructures. Using hydrogen with fuel cells could replace Li-ion batteries in drones and mobile gadgets as more efficient. Finally, some recent applications, including the potential use of Si-based agents as hydrogen sources to address issues associated with new approaches for antioxidative therapy. Hydrogen acts as a powerful antioxidant, specifically targeting harmful ROS such as hydroxyl radicals. Antioxidant therapy using hydrogen (often termed hydrogen medicine) has shown promise in alleviating the pathology of various diseases, including brain ischemia–reperfusion injury, Parkinson’s disease, and hepatitis.

## 1. Introduction

The utilization of fossil fuels for energy has led to numerous issues, including resource depletion and significant global environmental challenges. Due to its non-renewable nature, it is anticipated that the supply of fossil fuels will be largely exhausted by the year 2050 [1]. Moreover, the emission of carbon dioxide has caused severe environmental problems, notably contributing to the greenhouse effect [2]. As a result, renewable energy sources are receiving increased attention as viable alternatives to fossil fuels, owing to their perpetual availability and minimal environmental impact. Various renewable energy sources, such as solar power, wind, geothermal resources, and hydrogen, are being explored. Among these, hydrogen stands out as one of the most promising renewable energy sources due to its cleanliness and potential for sustainability [3,4,5,6].

Hydrogen boasts the highest gravimetric density among all fuels, indicating an exceptional energy density per unit mass. Its combustion releases energy in the form of heat, and when hydrogen interacts with oxygen in a fuel cell, the reaction produces energy in the form of electricity. Notably, unlike hydrocarbon-based fuels, both the combustion of hydrogen and its reaction with oxygen in a fuel cell occur without emitting greenhouse gases. This makes hydrogen a promising solution to address global warming concerns [7,8,9]. However, the challenge lies in its low volumetric density, resulting in lower energy density per unit volume, which makes the storage and transportation of hydrogen particularly challenging and costly. To facilitate the adoption of hydrogen as an energy carrier, it is essential to develop advanced storage methods capable of achieving higher energy density.

**Scheme 1:**
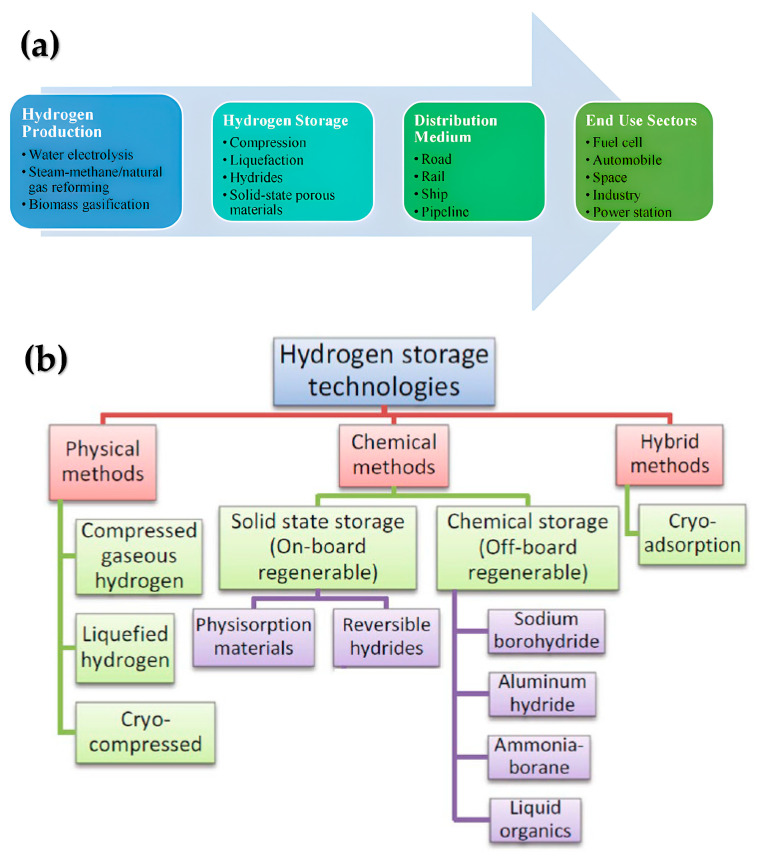
(**a**) schematic representation of the hydrogen energy economy [10], (**b**) hydrogen storage technologies [25].

The hydrogen economy encompasses production, storage, and transportation, all essential components for end-user applications, as depicted in Scheme 1a [10].

Currently, there are various methods for hydrogen gas production, including steam conversion of methane and natural gas, coal gasification, electrolysis of water, pyrolysis, partial oxidation, and more [11].

Below is a list of hydrogen production technologies aimed at efficiently producing hydrogen for various applications:

1. Thermochemical methods: hydrogen can be extracted from fossil fuels like coal, natural gas, and gasoline through thermochemical methods, with steam reforming being the most commonly employed [12,13]. In this process, fossil fuels react with steam using a nickel-based catalyst, releasing hydrogen. Additionally, hydrogen is derived from bio-oil obtained from biomass through the pyrolysis method, which also involves a reaction with steam.
2. Water electrolysis: this method involves using electrical energy to split water into hydrogen and oxygen, producing hydrogen gas [14,15,16].
3. Photoelectrochemical method: hydrogen is produced from solar energy through a process similar to electrolysis. Solar cells submerged in water generate electric current, making this process more efficient than conventional electrolysis [17,18,19].
4. Photobiological method: hydrogen production through natural photosynthesis activities of green algae, utilizing the photobiological method [20,21].
5. Chemical methods: hydrogen can be extracted from various hydride compounds, with sodium borohydride being a notable example [22,23,24].

Hydrogen storage technologies are illustrated in Scheme 1b [25], each method having unique characteristics. Existing hydrogen storage methods include liquefaction [26,27] and high-pressure tanks. However, these systems require extremely low temperatures or high hydrogen pressures [28], significantly increasing the costs and risks associated with hydrogen storage. Alternative approaches involve gas compression [29,30,31,32] and adsorption on highly porous materials such as carbon nanostructures, metal or complex hydrides, and metalloorganic networks [33,34,35,36,37,38,39]. While hydrogen can be stored, transported, or utilized in liquid or gaseous form, gas storage demands substantial quantities of energy. Up to 15% of the stored hydrogen energy is expended on compression.

**Figure 1:**
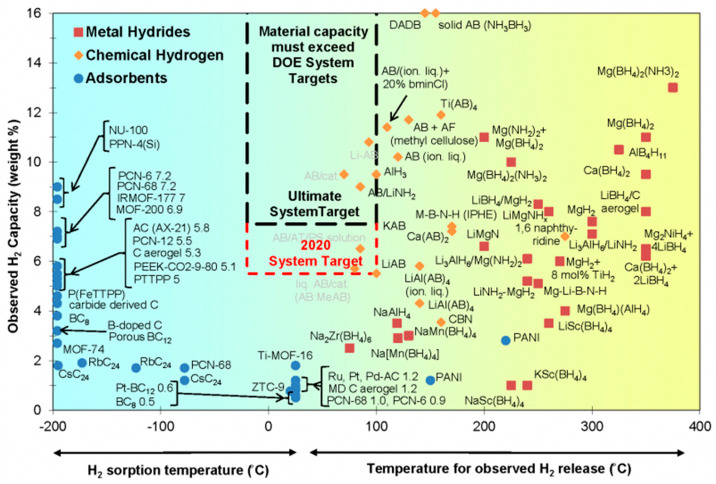
The hydrogen gravimetric capacity as a function of the hydrogen release temperature for numerous unique hydrogen storage materials [39].

The Department of Energy (DOE) regularly offers forecasts regarding the utilization of hydrogen energy. This includes taking into account promising materials for hydrogen storage that possess a high gravimetric hydrogen content within the material. As depicted in Figure 1, the hydrogen gravimetric capacity is presented as a function of the hydrogen release temperature for many of the unique hydrogen storage materials. It is evident that currently, research is being conducted on metal hydride, chemical hydrogen storage, and sorbent materials.

For metal hydride materials, the focus is on enhancing volumetric and gravimetric capacities, as well as hydrogen adsorption/desorption kinetics, cycle life, and reaction thermodynamics [40]. In the research of chemical hydrogen storage materials, the objectives involve increasing capacity, enhancing transient performance, reducing volatile impurities, and developing regeneration processes. The research on sorbent materials aims to increase the effective adsorption temperature and improve storage capacities through various means such as optimizing pore size and increasing pore volume and surface area [41].

Silicon (Si), on the other hand, is a promising candidate for hydrogen storage due to its natural abundance, cost-effectiveness, and favorable interaction with hydrogen [42,43,44]. Although the affinity of a pure silicon surface for hydrogen is limited, with reported adsorption energies of about −4.28 kJ·mol^−1^ [45,46], Si–Si bonds remain intact as hydrogen atoms form dangling bonds with surface silicon. Theoretically, for x = 1, 2, and 3, the weight percent (wt%) of hydrogen in a SiHx system is calculated to be 3.44, 6.65, and 9.65, respectively [36], and several storage systems incorporating porous media and related structures have been reported [45,47]. In this context, it is important to note that hydrogen storage can proceed via physisorption (weak van der Waals interactions, ~4–10 kJ·mol^−1^, efficient only at low temperatures), chemisorption (stronger covalent/ionic bonds, ~40–100 kJ·mol^−1^, requiring higher release temperatures), or a combination of both [48,49,50]. Porous silicon frequently exhibits this hybrid behavior, where hydrogen is bound through surface Si–H groups and simultaneously physisorbed within pores, enabling uptake and release under near-ambient conditions [51,52,53,54]. This dual mechanism provides a useful framework for comparing different storage strategies and for understanding the specific interactions that govern the performance of silicon-based systems.

Porous Silicon (PS) demonstrates significant potential as a highly efficient solid-state hydrogen storage material [25,55]. Electrochemical etching creates a surface on PS covered with SiH_x_ groups, giving it the capability to function as a solid-state hydrogen reservoir [56]. Although PS is a promising candidate for hydrogen storage, ongoing research aims to further enhance its efficiency [42]. For example, one study investigates the effectiveness of doping the pores to enhance performance [57]. Existing methods relying on chemically bonded hydrogen typically demand high temperatures for hydrogen extraction. Meanwhile, materials based on physisorption, which utilize Van der Waals forces, can only accumulate hydrogen at low temperatures to prevent desorption. However, nanosilicon and porous silicon offer a solution to this problem. Both the accumulation and extraction of hydrogen can take place at room temperature with these materials. Moreover, the use of this material can result in a high yield of hydrogen, thanks to both the chemical bonds (Si-H)_n_ and the catalytic properties of nanosilicon.

It should be emphasized that hydrogen generation from silicon nanostructures in aqueous environments is driven by chemical surface reactions, catalytic effects, and adsorption–desorption processes. Surface Si–H groups oxidize to form Si–O bonds with the release of hydrogen, while the high surface area and structural defects of nanosilicon enhance reactivity. Surface modifications such as doping further lower activation barriers and accelerate hydrogen evolution. In porous silicon, hydrogen can also be stored through physisorption within pores, with uptake and release possible under ambient conditions. The combined effects of surface chemistry, catalytic activity, and porous architecture make silicon nanomaterials efficient platforms for hydrogen generation and storage under mild conditions.

The development of Si-based agents as pharmaceuticals presents several challenges. Numerous reports highlight hydrogen generation from silicon nanoparticles [58,59]. These Si-based agents, stemming from interdisciplinary research, represent novel antioxidant substances derived from inorganic precursors [36]. Additionally, they introduce a groundbreaking bioadministration method for the highly effective and selective antioxidant hydrogen. This innovative approach enables the sustained generation of large amounts of hydrogen in the intestinal tract following oral administration.

Antioxidant therapy is an effective strategy for treating diseases where oxidative stress contributes to symptom onset. The goal is to rapidly replenish the body’s depleted antioxidant levels caused by excessive oxidative stress. It is crucial for a supplemented antioxidant to selectively eliminate harmful reactive oxygen species (ROS) while sparing physiologically beneficial ROS essential to the body. Conventional antioxidant therapies, while effective, can lead to adverse effects due to their lack of specificity.

Koyama’s review [60] suggests that Si-based agents are groundbreaking drugs capable of overcoming these challenges associated with current antioxidant therapy. These agents alleviate symptoms of oxidative stress-related diseases by generating substantial amounts of the antioxidant hydrogen in the body. Moreover, Si-based agents are anticipated to be highly effective therapeutic candidates, possessing anti-inflammatory, anti-apoptotic, and antioxidant effects.

This review aims to provide a comprehensive summary of the characterization and properties of silicon nanoparticles, porous silicon (PS), and silicon nanowires concerning their applications in hydrogen generation and storage. Additionally, it delves into the therapeutic effects associated with the antioxidant hydrogen produced by silicon nanoparticles.

## 2. Hydrogen Generation from Silicon Nanostructures

### 2.1. Hydrogen Generation from a-Si:H Nanostructures

**Figure 2:**
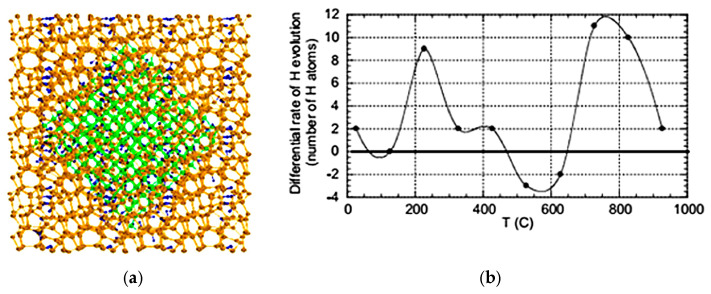
(**a**) Structure of the 4059-atom heterogeneous a-Si:H showing a nano-crystallite of c-Si within a matrix of a-Si:H. (**b**) The differential number of H released as a function of temperature obtained by the difference in H released found at each successive temperature [64].

Although extensive experimental studies have explored hydrogen generation from silicon nanomaterials and its underlying mechanisms, computational modeling remains relatively limited [61,62,63,64]. In this context, molecular dynamics simulations have been employed to investigate the temperature-dependent evolution of hydrogen from nanocrystalline silicon [65]. The model used in these simulations consisted of nanocrystallites heterogeneously dispersed within an amorphous silicon matrix (Figure 2a), with results indicating an excess hydrogen density localized at the nanocrystallite surfaces. A two-step hydrogen evolution process was observed, with a low-temperature peak between 250 and 400 °C attributed to hydrogen release from the nanocrystallite surfaces, followed by a second, higher-temperature peak between 700 and 800 °C (Figure 2b). This two-peak behavior is consistent with experimental hydrogen evolution measurements in hydrogen-diluted amorphous silicon (a-Si:H) grown near the crystalline phase boundary. The findings suggest that hydrogen is released into mobile molecular and bond-centered-like configurations, providing valuable insights into the thermal behavior of hydrogen in silicon-based materials.

### 2.2. Hydrogen Generation from Si Nanoparticles

#### 2.2.1. Reactions of Si Nanoparticles with Water

The limited literature available on the subject [65,66,67,68,69,70,71] indicates that silicon holds promise as a material for hydrogen generation from water. It demonstrates stability, ease of transport, and, upon oxidation with water, can produce 14% of its own mass in hydrogen through overall reactions such as:

Si(s) + 4H_2_O(1) → Si(OH)_4_(l) + 2H_2_(g), (1)

Si(s) + 2H_2_O(1) → SiO_2_(s) + 2H_2_(g) (2)

the indices s, l and g denote the solid, liquid and gas phases, respectively.

Bulk silicon is well-known for its reaction with oxygen, which forms a uniform passivating oxide layer on its surface. In silicon nanoparticles (SiNP), the oxygen-to-silicon ratio increases as particle size decreases, due to the larger surface area-to-volume ratio. Consequently, because of their high surface area per volume, similarly to other nanoparticles used for hydrogen generation, silicon nanoparticles are expected to enable faster hydrogen production compared to bulk silicon [71].

#### 2.2.2. Size Dependance

**Figure 3:**
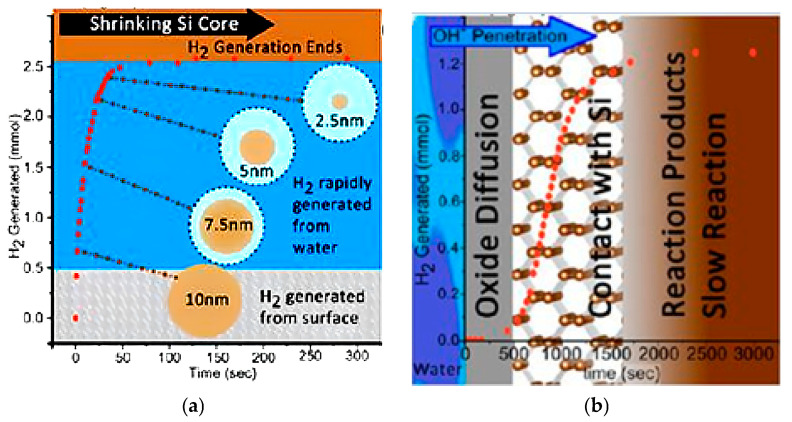
(**a**) Conceptual illustration of a shrinking core model that describes H_2_ generation from 10 nm Si NP. Brown spheres represent NPs, and the dotted circle indicates shrinkage from the original sphere size. The gray and blue areas represent hydrogen contributed by the silicon and by water, respectively. (**b**) A bulk diffusion model for reaction of larger particles (100 nm) for which OH^−^ diffuses through an oxide layer during the induction period and reaction rates remain limited by diffusion through reaction products [72].

Detailed studies have examined the influence of nanostructured silicon particle size on the efficiency of hydrogen evolution during interactions with aqueous solutions. To further explore this relationship, the size dependence of hydrogen generation has been investigated through base-catalyzed oxidation of silicon using particles of approximately 10 nm, sub-100 nm, and 325 mesh in diameter [72]. Various aqueous bases and reaction conditions, such as a Si/KOH molar ratio of 1:8, have been employed. Regardless of particle size and surface chemistry, all silicon samples eventually react with basic water, producing hydrogen. When reacting KOH in D_2_O with any of the Si particles, the resulting products were exclusively H_2_, HD, and D_2_O. The difference in etching behavior between 10 nm and 100 nm particles is evident when analyzing the apparent linear etching rate over time, as shown in Figure 3. The etch rate for 10 nm particles starts at approximately 0.3 nm/s and decreases linearly until it reaches zero when the particles are fully consumed. In contrast, the etch rate for 100 nm particles begins at zero, peaks at about 0.05 nm/s when roughly one-third of the silicon is consumed, and then decreases back to zero. This indicates that particle size influences the dynamics of the etching process and hydrogen production efficiency.

Goller documented the interaction of silicon nanocrystals with water/alcohol solutions, resulting in their complete oxidation, the hydrolysis of water, and the generation of hydrogen in quantities closely matching the ideal scenario [66]. Water acts as an oxidizer, while alcohol is essential for the efficient wetting of nanosilicon. This process culminates in the formation of a silicon-based alcogel, which can subsequently be transformed into an aerogel.

**Figure 4:**
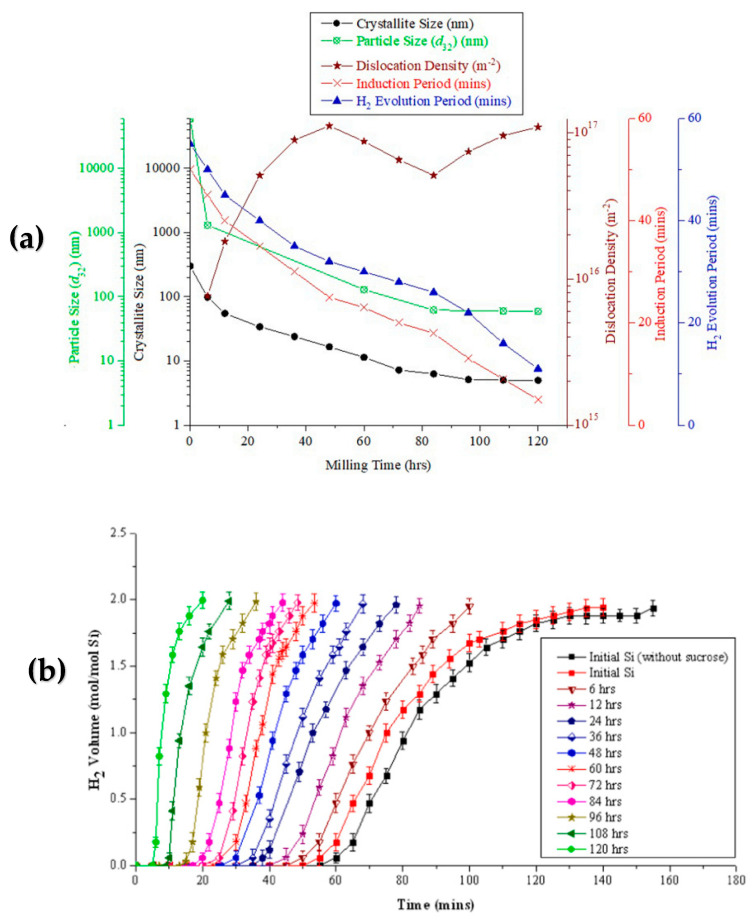
(**a**) Change in crystallite size and dislocation density with milling time; (**b**) H_2_ production over time during the reaction of initial 6, 12, 24, 36, 48, 60, 72, 84, 96, 108 and 120 h dry ball-milled Si samples mixed with sucrose (50% by weight) with 1 M NaOH alkaline water is shown. The same is shown for initial Si without sucrose for comparison [73].

The investigation of silicon powder for on-demand hydrogen production has yielded significant insights into its reactivity and structural evolution. Studies on the water-splitting properties of ball-milled silicon powder have examined the impact of microstructural parameters, such as crystallite size and dislocation density, on hydrogen generation efficiency (Figure 4) [73]. Systematic ball-milling for varying durations has revealed distinct trends in microstructural evolution, with crystallite size exhibiting a non-monotonic change before reaching saturation at approximately 84 h, while dislocation density continuously increased, doubling between 84 and 120 h of milling. Water-splitting experiments conducted in NaOH solution (pH 14) using ball-milled silicon powder mixed with 50 wt% sucrose demonstrated a notable enhancement in reactivity. The increased dislocation density was identified as a key factor contributing to a 2.8-fold increase in the maximum hydrogen generation rate. These findings underscore the potential of ball-milled silicon powder as an efficient material for hydrogen production, where microstructural modifications play a crucial role in optimizing its reactivity. However, it should be emphasized that the ball-milling approach is highly energy-consuming and inefficient for practical hydrogen production, which significantly limits its applicability despite the observed scientific insights.

#### 2.2.3. pH Dependance

Recent advancements in hydrogen generation using silicon nanopowder have demonstrated its potential as an efficient and scalable hydrogen source [74,75]. A bead milling approach has been developed for producing reactive silicon nanopowder, enabling hydrogen evolution even in near-neutral pH conditions (7.0–8.6). This method achieved a hydrogen generation rate of approximately 55 mL/g at pH 8.0 within one hour, corresponding to the hydrogen content found in approximately 3 L of saturated hydrogen-rich water [74]. These findings highlight the feasibility of using silicon nanopowder for hydrogen production under mild conditions, making it a promising candidate for practical applications.

**Figure 5:**
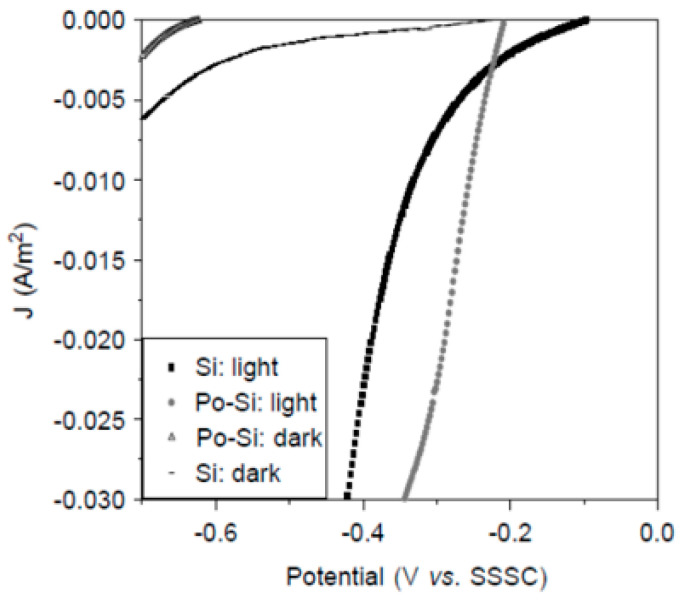
Current-potential characteristics in dark and light for Si and PS. The light flux was 150 mW/cm^2^ [76].

Further investigations have explored the influence of pH and temperature on hydrogen generation efficiency using silicon nanopowder derived from Si swarf [75]. The hydrogen evolution process was found to be strongly pH-dependent, with higher alkalinity leading to increased hydrogen production. At room temperature, the highest hydrogen yield of 1589 mL g^−1^ (corresponding to ~0.014 wt% H_2_) was observed at pH 13.9, while lower pH levels resulted in reduced hydrogen output. Additionally, an increase in reaction temperature to 50 °C significantly enhanced the hydrogen generation rate, from 122 mL min^−1^ g^−1^ (~0.001 wt% min^−1^) at pH 12.9 to 580 mL min^−1^ g^−1^ (~0.005 wt% min^−1^) at pH 13.0. Figure 5 illustrates the relationship between hydrogen volume and immersion time for Si nanopowder pre-treated with HNO_3_, demonstrating the effect of pH and temperature variations in KOH aqueous solutions. These findings underscore the importance of optimizing reaction conditions to maximize hydrogen production efficiency from silicon-based materials, while the gravimetric representation allows better comparison with other hydrogen storage approaches.

#### 2.2.4. Synthesis of Silicon Nanoparticles for Hydrogen Gas Production

The variety of methods highlights the adaptability of Si NP synthesis techniques to meet specific requirements of size, purity, and functionalization. Among these approaches, microwave-induced decomposition of silane has been employed to produce high-purity Si single crystals with particle sizes ranging from 6 nm to 11 nm, demonstrating a scalable route for generating monodisperse nanoparticles [76]. Additionally, a two-step method involving nanosecond laser ablation of a Si wafer in chloroform (CHCl_3_), followed by ultrasonic treatment in a solvent mixture of isopropanol, hydrofluoric acid (HF), and hexane (3:1:3), has been employed to synthesize small-sized Si NPs [77]. By integrating laser and ultrasonic processes, this technique provides a versatile and accessible route for nanoparticle production using widely available reagents.

Beyond synthesis, advancements in Si NP functionalization have further expanded their potential applications, particularly in biomedical fields. For instance, anionic porphyrin-grafted porous Si nanoparticles with sizes ranging from 35 nm to 245 nm have been synthesized through a two-step modification process [78]. The first step involved hydrosilylation with allylisocyanate at 90 °C for 2 h, followed by covalent grafting of porphyrin-NH_2_ through an amine linkage at 80 °C for 18 h. This functionalization strategy enhances the biocompatibility of Si NPs, enabling their use in targeted drug delivery, particularly for porphyrin-based cancer therapies. Collectively, these synthesis and modification approaches underscore the versatility of Si NP fabrication, offering tunable structural and surface properties for diverse technological and biomedical applications.

**Figure 6:**
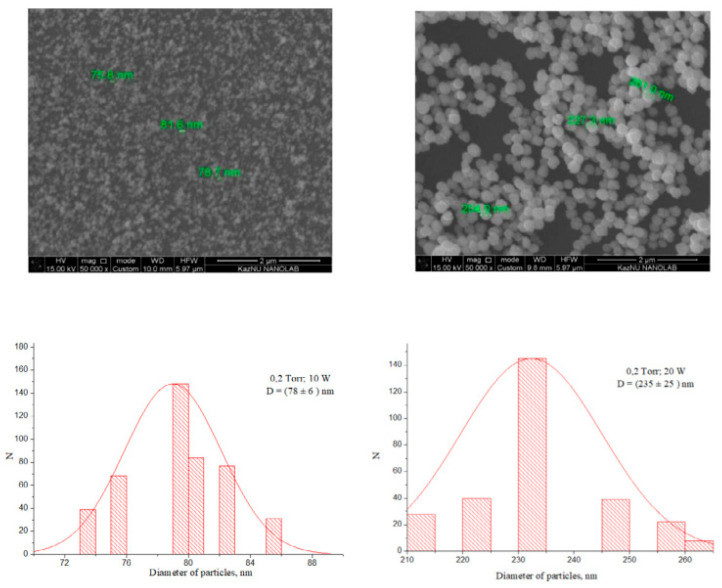
SEM images of synthesized nanoparticles at gas pressure p = 0.2 Torr and different discharge power. And diameter distribution graphs [79].

Further expanding the range of Si NP synthesis techniques, plasma-assisted methods have been explored as an alternative approach for nanoparticle fabrication. The synthesis of silicon nano- and microparticles has been successfully achieved in the plasma of an RF discharge in a monosilane (2%) and argon (98%) mixture, with pressure, power, and synthesis time serving as key parameters influencing particle formation and growth [79]. Experimental findings indicate that discharge power and gas pressure play crucial roles in controlling nanoparticle size, enabling the production of Si NPs with diameters below 100 nm. By precisely adjusting plasma conditions, Si NPs ranging from 50 nm to 100 nm can be synthesized, making them particularly suitable for hydrogen production (Figure 6). These studies demonstrate how different synthesis methods can be used to tailor Si NP properties, making them more suitable for various technological and biomedical applications. Controlling particle size, surface chemistry, and functionalization enhances their potential for targeted uses, from drug delivery to sustainable energy solutions.

### 2.3. Hydrogen Generation from Porous Silicon

#### 2.3.1. Porous Silicon

The widely recognized method for producing porous silicon (PS) involves anodic etching in an HF:C_2_H_5_OH electrolyte. This anodization process yields PS that contains a substantial amount of chemically bound hydrogen. The chemical composition of nano-PS produced at high current densities is represented by SiH_x_, where x ranges from 1 to 2 [80]. This method not only yields PS with a significant hydrogen content but also creates a fractal-like surface entirely covered by SiH_x_ bonds [81]. The presence of hydrogen significantly influences the physical properties of the Si nanocrystallites in PS nanostructures. Hydrogen coverage drives the expansion of nanocrystallites [82], which in turn alters their electronic properties [83]. This indicates that controlling hydrogen content can be a key factor in tuning the physical and electronic properties of PS.

Extensive research on PS nanostructures has demonstrated their potential as efficient hydrogen storage reservoirs, with studies examining both storage capacity and underlying mechanisms. The high hydrogen content of PS, combined with its unique surface chemistry, enhances its ability to store and release hydrogen efficiently [65,84,85,86]. These properties position PS as a promising material for advanced hydrogen storage systems, where controlled molecular-level interactions are crucial for achieving high-capacity and reversible hydrogen storage.

#### 2.3.2. Hydrogen Desorption from Porous Silicon via TPD Technique

The electrochemical etching process results in the formation of PS with a surface densely populated with hydrogen-terminated species, leading to significant hydrogen coverage [87]. Fourier transform infrared (FTIR) spectroscopy has been extensively used to analyze the chemical composition of PS, revealing the presence of both SiHx species and oxidized surface groups that form during thermal annealing [88,89]. These detailed spectral assignments provide crucial insights into the interaction of PS with hydrogen and oxygen, contributing to a deeper understanding of its surface chemistry.

Further investigations utilizing temperature-programmed desorption (TPD) have demonstrated the release of hydrogen from n-type PS, although the precise hydrogen storage capacity remains undetermined [90]. In contrast, a combined FTIR and TPD analysis of p^+^-type PS has provided a quantitative estimation of hydrogen desorption, indicating that approximately 2 mmol/g of hydrogen is released within the 40–850 °C temperature range [91]. These findings suggest that p^+^-PS surfaces are predominantly hydrogen-terminated, with minimal exposure of Si atoms as adsorption sites. A comparative analysis of FTIR and TPD mass spectrometry data confirms that the majority of desorbed hydrogen originates from surface-bound SiH_x_ species, emphasizing the critical role of surface chemistry in governing hydrogen storage and release properties.

Understanding the chemical composition and thermal behavior of PS, as revealed through FTIR and TPD studies, is essential for optimizing its performance in hydrogen storage applications. The identification of SiH_x_ and oxidized species provides valuable insights into the stability, reactivity, and potential functionalization of PS under various conditions, further supporting its viability as a hydrogen storage material.

#### 2.3.3. Porous Silicon as Photocathode in Hydrogen Production

Solar energy is a prominent renewable resource with the potential for producing fuels, chemicals, and generating power. Among the methods for solar hydrogen production, thermochemical production [92] and photoelectrolysis [93,94] have garnered substantial attention. Photoelectrolysis of water using semiconductor materials is particularly fascinating as it involves the creation of electron-hole pairs upon light irradiation, which are then utilized to decompose water into hydrogen and oxygen. Numerous studies have explored photoelectrochemical hydrogen production using silicon [95,96,97].

**Figure 7:**
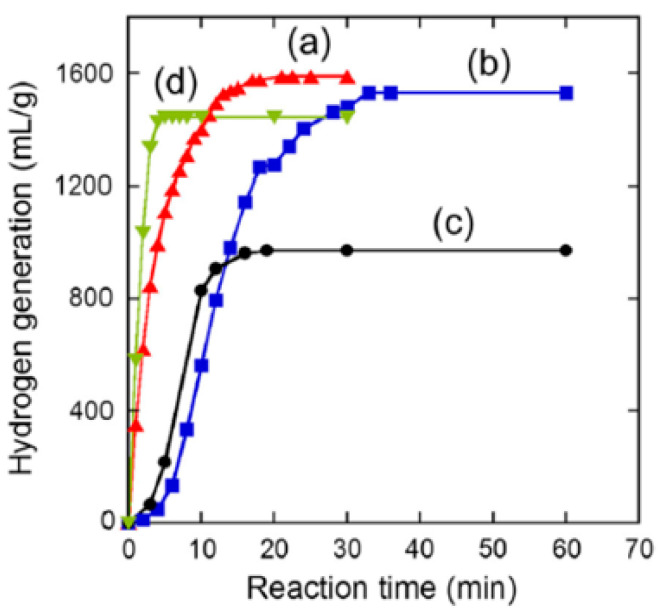
Volume of hydrogen generated by the reaction of Si nanopowder with KOH aqueous solutions having following pH values and temperatures versus the reaction time: (a) pH 13.9, RT; (b) pH 13.4, RT; (c) pH 12.9, RT; (d) pH 13.0, 50 °C [98].

PS has been investigated as a promising material for enhancing hydrogen production in PEC systems, leveraging its increased surface area and reactivity compared to bulk silicon [98]. Electrochemical characterization of Si and PS photocathodes under both dark and illuminated conditions reveals a significant enhancement in cathodic current for PS, indicating a superior hydrogen evolution performance. In contrast, negligible photocurrent generation at the Si photocathode suggests that unmodified silicon is not an efficient material for PEC water splitting (see Figure 7). Furthermore, PS-based photocathodes exhibit stable performance in acidic environments over multiple measurements, demonstrating their durability under operational conditions.

The dependence of hydrogen evolution potential on light intensity has been systematically examined, showing a linear correlation between hydrogen production rates and photon flux. These findings underscore the importance of surface engineering and material modifications in optimizing silicon-based photoelectrodes, further advancing the development of efficient PEC systems for sustainable hydrogen production.

#### 2.3.4. Mesoporous Silicon Spheres for Photocatalytic Hydrogen Evolution

The efficiency of particulate silicon in photocatalytic H_2_ production is primarily limited by its energy band configuration and surface characteristics. The proximity of silicon’s conduction band edge to the water reduction potential limits its ability to produce H_2_ efficiently with low-energy photons [99]. A photoelectrochemical (PEC) approach is often employed for silicon photoelectrodes to address this issue, which involves bending the energy bands with an applied bias [100,101]. However, this PEC method usually requires a conductive substrate and an external bias, adding complexity and increasing production costs [102]. Nanoscaling is a promising strategy to widen the energy gap between the conduction band edge and the water reduction potential, thus overcoming the energy band limitation in H_2_ production through quantum confinement [103]. Another challenge is the limited number of active sites on the surface of silicon photocatalysts. Creating porous structures can mitigate this issue by providing a larger specific surface area and enhancing light trapping through multiple reflections within the pores [104,105]. This approach increases the number of active sites available for the photocatalytic reaction, thereby improving efficiency.

**Figure 8:**
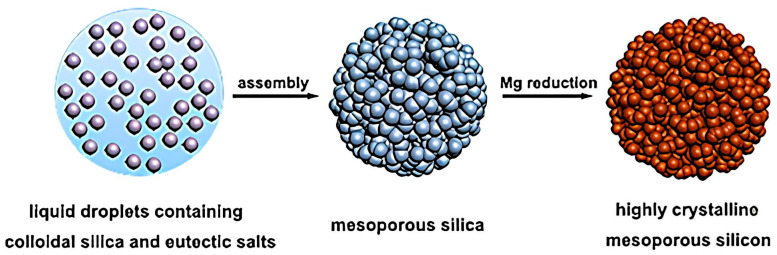
Schematic illustration for the synthesis of highly crystalline mesoporous silicon spheres (CMSSs) [106].

A novel and efficient approach has been developed for the synthesis of highly crystalline mesoporous silicon spheres (Figure 8) using a salt-assisted aerosol process combined with magnesiothermic reduction [106]. This method offers a significant advantage by eliminating the need for toxic etching agents and hazardous metal reductants, making it a safer and more sustainable alternative for silicon nanomaterial fabrication. By precisely tuning the synthesis conditions, the porous structure can be controlled, enabling modifications in bandgap, light absorption, charge separation, and surface reactivity, all of which are critical for enhancing photocatalytic performance. The resulting silicon spheres exhibit exceptional and stable photocatalytic hydrogen production under visible light irradiation, achieving an impressive rate of 1785 mmol h^−1^ g^−1^, surpassing previously reported porous silicon materials. This enhanced efficiency underscores the potential of such materials for solar-driven hydrogen generation, offering a scalable and practical route for sustainable fuel production. The ability to harness solar energy for hydrogen production through photocatalysis represents a crucial step toward environmentally friendly energy solutions. Further advancements in silicon nanomaterial synthesis and photocatalytic optimization will play a pivotal role in the development of efficient solar fuel technologies, addressing the growing demand for clean and renewable energy sources.

#### 2.3.5. Stain Etching of Porous Silicon

For large-scale industrial production of PS, the development of electroless technology is necessary. The findings from Litvinenko et al. [65] underscore the potential of stain etching as a scalable and efficient method for producing porous silicon for hydrogen storage and production. This method converts each particle of crystalline silicon into a particle with a porous shell and a crystalline core. From an industrial perspective, stain etching is more appealing than electrochemical etching as it eliminates the need for an external electrical power source. The etching solution comprised H_2_O, HF, and HNO_3_ in a ratio of 20:4:1 [100], with the etching process lasting between 10 and 60 min, depending on temperature and HNO_3_ concentration. The reaction was halted when the powder turned brown, and careful attention was paid to prevent the dissolution of the newly formed porous shell. The resulting foam product was collected, rinsed with water and ethanol, and dried under ambient conditions.

**Figure 9:**
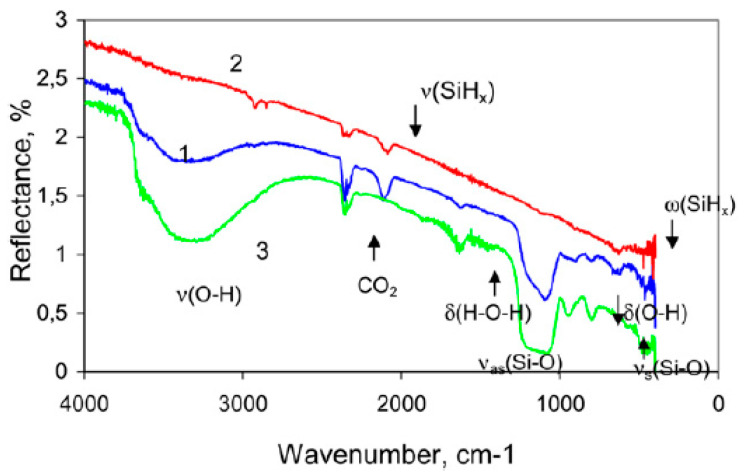
DRIFT spectra of PS samples: PS_init is the PS, obtained from metallurgical grade Si powder, PS_HF/EtOH is the PS_init treated by ethanolic HF (5%), PS_NH_3_ is the PS_init treated by NH_3_ in H_2_O [65].

Treating stain-etched PS with aqueous NH_3_ solutions results in hydrogen release and the creation of hydrated SiO_2_. This is evidenced by reaction (3) and supported by the complete disappearance of SiH_x_ bands and the increased presence of Si-O bands in the DRIFT spectra (see Figure 9).

SiH_x_ + H_2_O(NH_3_) → H_2_SiO_3_ + (2 + x/2)H_2_↑, (3)

### 2.4. Hydrogen Generation from Silicon Nanowires

#### 2.4.1. Reaction of Silicon Nanowires with Water

PS encompasses a wide range of nanostructured morphologies, including silicon nanowires (SiNWs), which represent a distinct class of elongated, high-aspect-ratio porous architectures. These structures retain the high surface area and tunable porosity characteristic of PS while exhibiting unique mechanical and electronic properties that enhance their reactivity and functionality [107].

Ultralong porous SiNW arrays, approximately 400 μm in length [108], have been successfully synthesized using the metal-assisted anodic etching (MAAE) method [109,110,111,112]. The fabrication process allows precise control over the diameter and density of the SiNW arrays through nanosphere lithography, enabling the formation of structures optimized for specific applications [109,110,113,114]. The resulting SiNWs measure 425 ± 25 µm in length and 200–300 nm in diameter, exhibiting a porous architecture with irregular edges and surfaces. These nanowires possess a high specific surface area of 413 m^2^/g and an average pore diameter of 4.6 nm, which significantly enhances their reactivity.

**Figure 10:**
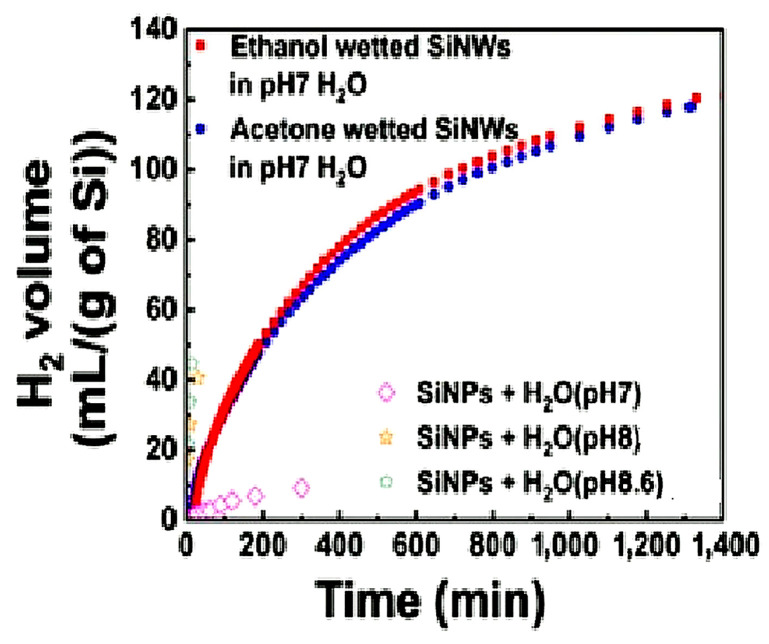
The total amount of generated H_2_ volume in mL/(g of Si) vs. time measured by gas chromatography (GC) for SiNWs [74,108].

A remarkable feature of these porous SiNW arrays is their ability to undergo a spontaneous reaction with water, generating hydrogen under ambient and dark conditions without requiring external energy input (Figure 10). The observed hydrogen evolution rate is approximately ten times higher than previously reported values for other silicon nanostructures. This enhanced reactivity has been attributed to two primary factors: the high specific surface area, which prevents nanowire agglomeration, and the intrinsic strain within the SiNWs, which facilitates water-mediated oxidation by promoting Si–Si bond cleavage. These findings highlight the potential of porous SiNW arrays as a highly efficient material for sustainable hydrogen generation, leveraging their unique structural and chemical properties.

Building on these advancements, SiNWs have also been integrated into photocathode assemblies for enhanced photoelectrocatalytic hydrogen evolution. In one such approach, a porous photocathode was developed utilizing SiNWs as a light absorber and nonprecious molybdenum sulfide (MoS_x_) as a hydrogen-evolution catalyst [115]. The performance of this system was largely influenced by the diameter of the SiNWs, which was systematically tuned between 13 and 48 nm to optimize the light absorption range. A simple and effective method was demonstrated for the direct synthesis of SiNWs with controlled diameters on a porous conductive support, ensuring high quality and uniformity. This level of precision in fabrication provided unprecedented insights into the SiNW growth process, suggesting a silylene-based mechanism. The SiNW/MoS_x_ photocathodes exhibited stable hydrogen evolution under illumination for several hours, maintaining a faradaic efficiency exceeding 98%. These findings underscore the potential of SiNW-based photocathodes for highly efficient and scalable solar-driven hydrogen production, offering a promising route toward sustainable energy conversion technologies.

#### 2.4.2. SiNW Arrays with Cobalt Phosphide for Efficient Solar Hydrogen Evolution

Silicon microwire (SiMW) and silicon nanowire (SiNW) have been explored as photocathodes for the hydrogen evolution reaction (HER) using techniques like reactive ion etching, chemical vapor deposition (CVD), and metal-assisted chemical etching (MACE) [115]. Bare silicon is known for its slow kinetics in HER, usually generating minimal photocurrent at 0 V versus the reversible hydrogen electrode (RHE), where hydrogen evolution thermodynamically begins [116]. This limitation necessitates the use of a co-catalyst to enhance the HER performance of the electrode. Platinum (Pt) is the most effective and commonly used co-catalyst for SiMW/SiNW photocathodes, but its high cost and limited availability make it impractical for large-scale applications.

**Figure 11:**
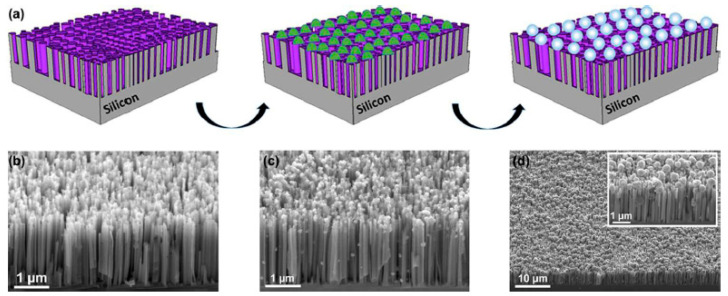
(**a**) Schematic illustration of the fabrication of Co-P sphere decorated SiNW arrays. Representative SEM micrographs of (**b**) the as-obtained SiNW arrays; (**c**) SiNW arrays coupled with photo-electrodeposited Co NPs, and (**d**) SiNW/Co-P-500 prepared by a phosphorization treatment at 500 °C for 6 h in high-purity N_2_ [116].

An advanced strategy has been developed for enhancing HER performance by integrating SiNW arrays with hollow cobalt phosphide (Co–P) spheres, an earth-abundant and highly efficient catalyst [116]. These SiNW/Co–P photocathodes exhibit a significantly more positive onset potential (V_onset_), defined as the potential at which the cathodic current density reaches 1 mA cm^−2^, compared to unmodified SiNW arrays (see Figure 11). Additionally, they achieve a substantially higher photocurrent density at 0 V versus the reversible hydrogen electrode (RHE), indicating enhanced charge transfer and catalytic efficiency. The photoelectrochemical performance of SiNW/Co–P electrodes are comparable to that of SiNW arrays decorated with electrolessly deposited platinum nanoparticles (SiNW/PtNPs), highlighting the potential of cobalt phosphide as a viable alternative to precious metal catalysts. These findings reinforce the role of hybrid SiNW-based photocathodes in advancing scalable and cost-effective HER technologies, offering an efficient pathway for sustainable solar-driven hydrogen production.

Among non-precious catalysts, cobalt phosphide stands out due to its elemental abundance on Earth, making SiNW arrays decorated with cobalt phosphide a highly promising and cost-effective option for photocathodes in water-splitting cells. This combination not only addresses the cost and availability issues associated with platinum but also offers competitive performance, making it a viable alternative for sustainable hydrogen production.

The research highlights the potential of silicon nanowire arrays, particularly when combined with cobalt phosphide, as effective and economical photocathodes for hydrogen evolution reactions. This advancement could significantly contribute to the development of cost-efficient and scalable solutions for solar fuel production, leveraging abundant materials while maintaining high performance. The success of SiNW/Co-P photocathodes underscores the importance of exploring non-precious metal catalysts in enhancing the viability of water-splitting technologies.

#### 2.4.3. SiNWs with AgNPs for Hydrogen Generation

Recent advancements in SiNW-based photocatalysts have demonstrated the significant potential of silver nanoparticle (AgNP)-decorated SiNWs for hydrogen production in aqueous environments [117]. Highly doped n-type SiNWs, synthesized via a top-down fabrication approach, exhibit enhanced light-induced hydrogen evolution when modified with AgNPs under white light illumination. Comparative analysis revealed that AgNP-decorated SiNWs generate at least 2.5 times more hydrogen than their undecorated counterparts, highlighting the catalytic role of silver in boosting photocatalytic efficiency.

Spectroscopic investigations, including vibrational, UV–vis, and X-ray spectroscopy, indicate that the SiNW sidewalls are coated with silicon suboxides up to 120 nm thick. These suboxide layers exhibit wide bandgap semiconductor properties similar to titanium dioxide (TiO_2_), enhancing charge separation and promoting efficient photocatalysis. Notably, these coatings maintain structural and chemical stability during hydrogen evolution in water for at least three hours of continuous irradiation. Synchrotron-based studies further reveal that the increase in silicon’s bandgap results from the favorable positioning of the valence band in nanostructured silicon, which enhances the electronic properties of SiNWs. This band alignment facilitates efficient charge carrier dynamics, reinforcing the suitability of AgNP-decorated SiNWs for solar-driven hydrogen production. These findings suggest that the strategic incorporation of AgNPs into SiNWs could play a key role in developing high-performance and stable photocatalytic systems for sustainable hydrogen generation.

In summary, we can conclude that most silicon-based nanomaterials contribute to hydrogen generation through two distinct processes. The irreversible reaction of Si with water leads to hydrogen release via surface oxidation, but consumes the silicon material and is therefore non-reversible [65,86,118]. In contrast, the photocatalytic route relies on photoexcited carriers in Si nanostructures to drive water splitting, offering the potential for continuous hydrogen evolution under illumination. Although both are often reported under the general term “hydrogen generation,” they differ fundamentally in mechanism and reversibility. Moreover, while the mechanisms of hydrogen release in the Si–H_2_O reaction have been extensively investigated and are relatively well understood, the situation with photocatalytic processes remains less clear: only a few theoretical works have attempted to provide a more detailed picture of the underlying pathways [119,120].

### 2.5. Silicon Based Nanohybrids for Enhanced Generation of Hydrogen

#### 2.5.1. Nanosilicon with Grafted WS_2_

One of the most promising technologies for hydrogen production today is water electrolysis. Platinum-based catalysts have been the primary electrocatalysts for the HER, but their high cost and limited availability pose significant barriers to their widespread adoption. Nano-silicon has emerged as a successful alternative in developing new electrocatalysts [121].

Recent investigations have explored the integration of tungsten disulfide (WS_2_) with silicon and silicon nanoparticles to enhance electrocatalytic performance [122]. In this approach, WS_2_ is deposited onto Si and nano-Si substrates, followed by chemical etching, which modifies the structural and electronic properties of the material. The presence of WS_2_ serves as an effective catalytic site for HER, while silicon nanoparticles act as a support, facilitating charge transfer and improving overall electrochemical activity. The study utilized electrochemical impedance spectroscopy, linear sweep, and cyclic voltammetry to assess the electrochemical properties of WS_2_/Si and WS_2_/n-Si before and after chemical etching.

The results demonstrated that the WS_2_/nano-Si/etched exhibited high electrocatalytic activity, characterized by high current density, low overpotential for HER (0.14 V), small Tafel slopes (45 mV dec^−1^), and large cathodic currents. These findings indicate that the chemical etching process of silicon in HF significantly increased the number of active sites, thereby enhancing the electrocatalytic behavior of the material. The study underscores the importance of silicon nanoparticles in improving the electrocatalytic properties of tungsten disulfide and demonstrates that chemical etching can significantly enhance the activity of the catalyst by increasing the number of active sites.

#### 2.5.2. Silicon–Carbon Composites Modified with Nickel and/or Platinum

The study by Elsodany et al. [123] demonstrates that the combination of platinum and nickel within a carbon–silicon matrix significantly improves the electrocatalytic performance for the hydrogen evolution reaction. Composites of modified silicon, including C–Si, Ni/(C–Si), Pt/(C–Si), and Pt–Ni/(C–Si), were prepared and evaluated as electrocatalysts for the HER in a 0.5 M H_2_SO_4_ solution. The incorporation of Pt in a small ratio with Ni into [C–Si (1:1)] enhances the stability and activity of the electrode due to the synergistic interaction between the Ni–Pt bimetallic species. Tests conducted on the as-prepared composite electrodes revealed that the [7%Pt–3%Ni]/[C–Si (1:1)] composite exhibited the highest activity and stability towards the HER in an acidic medium compared to other composites in the study. This composite showed the lowest overpotential (0.358 V vs. Ag/AgCl, approximately 0.161 V vs. RHE) at 10 mA cm^−2^, the best exchange current density (5.345 mA cm^−2^), and the lowest charge transfer resistance (2.657 Ω). Additionally, it achieved the highest turnover frequency (TOF) value of 4.219 × 10^−3^ s^−1^ and a mass activity of 642 mA g^−1^, outperforming other composites.

Furthermore, the composite demonstrated good stability for the hydrogen evolution reaction, with the current density reaching about 34.28 mA cm^−2^ after 5 h of operation. This research highlights a viable pathway for developing cost-effective and efficient electrocatalysts for green hydrogen production. By leveraging the synergistic properties of bimetallic composites, this approach could contribute to the advancement of sustainable energy technologies, reducing dependence on traditional fossil fuels and promoting the use of renewable energy sources.

## 3. Hydrogen Storage in Silicon Nanostructures

### 3.1. Modeling Metal-Decorated Porous Silicon for Hydrogen Storage

**Figure 12:**
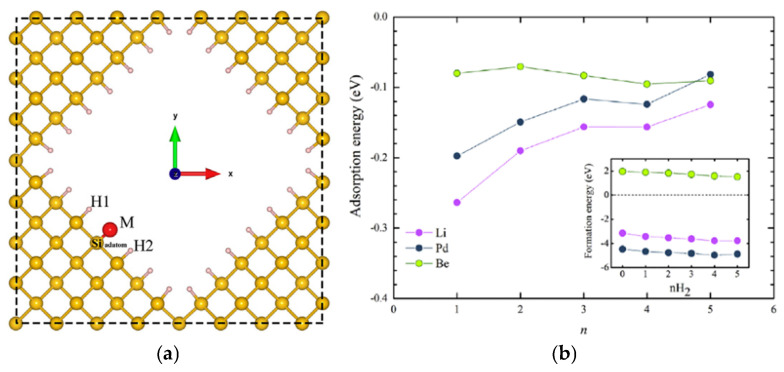
(**a**) Cross-section of the hydrogenated PS with one adatom. (**b**) Adsorption energy per H_2_ molecule and formation energy (inset) evolution with respect to the number of H_2_ molecules adsorbed, on the Li-decorated (magenta), Be-decorated (green) and Pd-decorated (blue) PS [124].

Investigations into the hydrogen storage potential of porous silicon (PS) have revealed that its performance can be significantly enhanced through atomic decoration with various elements, such as lithium (Li), beryllium (Be), and palladium (Pd) [124,125]. Despite the lack of theoretical investigations prior to this study, the experimental results offer a compelling look at the potential of PS as a solid-state hydrogen storage material. Li and Pd atoms show strong bonding with the pore walls of PS, while Be does not exhibit any bonding affinity. This difference in bonding behavior directly affects the hydrogen adsorption capabilities. Li and Pd can accommodate up to five and four H_2_ molecules, respectively, before the adsorption energy becomes too low or the molecules are positioned too far from the surface (see Figure 12). Be, on the other hand, does not facilitate H_2_ adsorption due to its lack of bonding with the pore walls. The strong chemisorption of Li and Pd to the pore walls enhances the physisorption of H_2_ molecules. The presence of passivating hydrogen atoms on the PS surface creates a region of effective charge by attracting electronic density from the metal. This charge region interacts favorably with the electronic density of H_2_ molecules, enabling efficient hydrogen storage. Among the atoms studied, Li emerges as the most effective for hydrogen storage. It offers robust chemisorption to the pore walls, favorable H_2_ adsorption energies, and the capacity to store the maximum number of hydrogen molecules. Additionally, Li’s lighter atomic weight provides an added advantage.

PS stands out due to its large surface area and ease of synthesis compared to other materials, such as atomically thin monolayers. While these monolayers might have higher storage capacities, their synthesis methods are still underdeveloped, making PS a more practical option for current hydrogen storage applications.

The study highlights the importance of selecting appropriate decorating atoms to enhance hydrogen adsorption properties. Porous silicon, with its substantial surface area and relatively simple synthesis process, presents a promising material for hydrogen storage. The findings underscore the potential of Li as a superior decorating atom, which could lead to more efficient hydrogen storage solutions. This research bridges the gap between experimental observations and theoretical investigations, providing a robust foundation for future studies and technological developments in hydrogen storage.

### 3.2. Hydrogen Storage in Porous Silicon Nanostructures

#### 3.2.1. Porous Silicon as a Hydrogen Reservoir

The hydrogen storage potential of porous silicon (PS) nanostructures has been extensively investigated, highlighting their exceptionally high internal specific surface area, which can reach up to ~430 m^2^ g^−1^ (equivalent to 1000 m^2^ cm^−3^) [80,126]. The surfaces of these nanostructures are predominantly covered with SiHx bonds [82]. Hydrogen has a notable impact on the physical properties of silicon nanocrystallites within PS nanostructures. For instance, hydrogen coverage is a key factor that causes the expansion of nanocrystallites, thereby altering their electronic properties [91]. A key aspect of PS nanostructures is their ability to release molecular hydrogen (H_2_) through the thermal decomposition of SiHx groups, which are uniformly distributed across the material’s surface [127,128]. This characteristic suggests that PS could serve as an effective hydrogen storage medium, offering a controllable release mechanism based on thermal activation.

Given the growing importance of hydrogen as a clean energy carrier, understanding the mechanisms governing hydrogen adsorption, retention, and desorption in PS nanostructures is essential for advancing hydrogen storage technologies and optimizing their practical applications.

#### 3.2.2. Properties of PS Suitable for H_2_ Storage: Porosity and Surface Area

**Figure 13:**
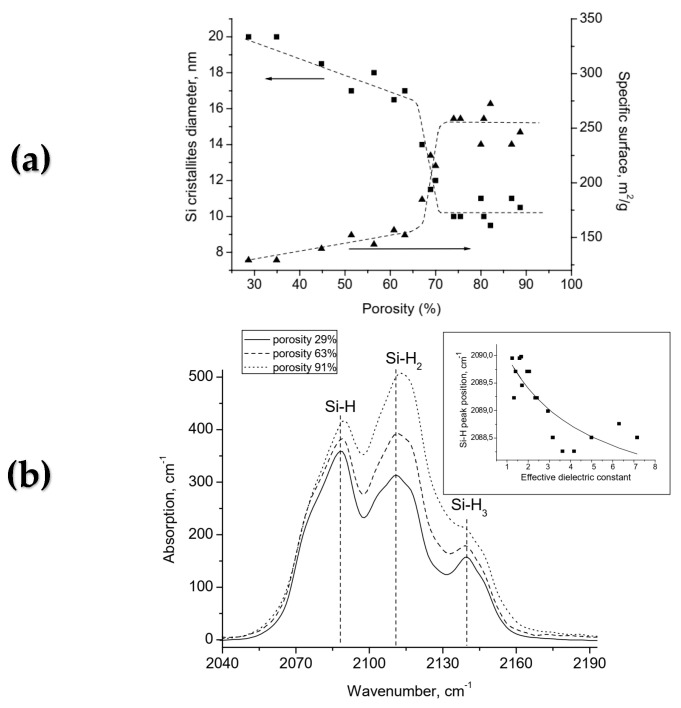
(**a**) Si nanocrystallites size (■) and specific surface (▲) of the meso-porous Si as functions of porosity. Dashed lines are added for eye guiding. (**b**) Infrared absorption spectra of as-prepared hydrogen terminated meso-porous Si for three porosity values. The insert shows Si–H peaks shift along with effective dielectric constant of the porous silicon [85].

The physical properties of PS, such as porosity, pore size, thickness, and specific surface area, are essential for its effectiveness as a hydrogen storage material. By adjusting etching parameters like HF concentration, current density, doping level, anodization duration, temperature, and wafer type, researchers can finely tune these properties to optimize PS for specific storage needs [112,128]. For instance, varying the HF concentration or current density during the etching process can significantly alter the porosity and surface area of the resulting PS, and adjusting the doping level or anodization duration can lead to variations in the pore structure and surface chemistry of PS, directly impacting its hydrogen storage efficiency. There is a direct correlation between porosity and surface area in mesoporous silicon. Higher porosity enhances the fractal nature of the surface, which is beneficial for hydrogen storage as it increases the availability of Si dihydride and monohydride bonds [129]. This relationship also highlights the importance of nano-crystallite size. As porosity increases, the nano-crystallite size typically decreases, leading to a larger surface area that can facilitate more hydrogen adsorption [55]. The graphical representation in Figure 13a provides a clear visual understanding of how surface area varies with porosity in mesoporous silicon. Such insights are invaluable for researchers aiming to optimize PS properties for hydrogen storage.

#### 3.2.3. Morphology Dependent SiH_X_ Spectral Features

The vibrational spectra of mesoporous silicon nanostructures provide valuable information about the Si-H bonds present in the material. In Figure 13b, the stretching vibration spectra of Si-H, Si-H_2_, and Si-H_3_ bonds are depicted, each represented by distinct bands centered at 2088 cm^−1^, 2110 cm^−1^, and 2137 cm^−1^, respectively [85]. These bands indicate the presence of different Si-H configurations in the nanostructures. The analysis of these spectra underscores the evolution of bonding configurations with changing porosity, emphasizing the importance of porosity and surface roughness in influencing the hydrogen storage efficiency of mesoporous silicon.

The evolution of Si-H and Si-H_2_ peak intensities with varying porosity levels reveals a significant relationship between porosity and hydrogen bonding configurations. At lower porosity levels (<63%), the dominance of the Si-H peak suggests a prevalence of monohydride bonds. As porosity increases beyond 63%, the Si-H_2_ peak becomes more prominent, indicating a shift towards dihydride bonding. This balance between monohydride and dihydride bonds at intermediate porosity (~63%) highlights the dynamic nature of hydrogen bonding as a function of structural changes in the nanomaterial.

**Figure 14:**
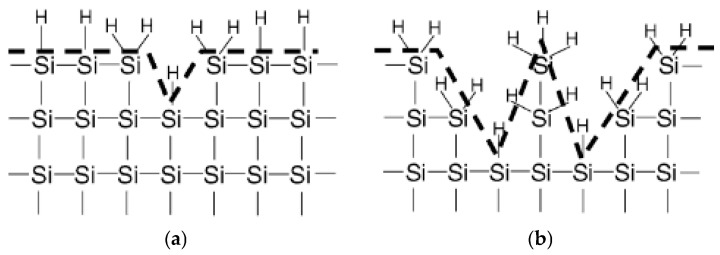
Schematic view of porosity dependent fractal-like roughness of Si nanocrystallites surfaces at atomic level: (**a**) low porosity (<60%); (**b**) high porosity (>60%). Dashed line points the nanocrystallites surfaces [85].

The increase in surface roughness with higher porosity levels significantly influences the hydrogen bonding configurations on the Si nanocrystallite surface. At lower porosities, smoother surfaces limit the formation of SiH_2_ bonds due to fewer available Si atoms that can bond with two hydrogen atoms (Figure 14a). As the surface roughness increases with porosity, more Si atoms are exposed and available to form SiH_2_ bonds, enhancing the material’s capacity for hydrogen storage (Figure 14b) [85]. This relationship between surface roughness and hydrogen bonding underscores the importance of optimizing surface features to maximize storage efficiency.

The intricate relationship between porosity, surface roughness, and hydrogen bonding configurations highlights the need for precise control over the structural properties of mesoporous silicon. By fine-tuning these properties, it is possible to enhance the hydrogen storage capabilities of the material. The fractal nature of the Si nanocrystallite surface, as suggested by Lysenko et al. [85], plays a crucial role in determining the bonding configurations and, consequently, the hydrogen storage efficiency. Understanding these relationships is key to developing more effective hydrogen storage materials.

#### 3.2.4. Hydrogen Concentration and Nanocrystallites Dimensions in Porous Silicon

Nano-PS (diameter < 7 nm, surface area 200–400 m^2^/g) and meso-PS (diameter 7–50 nm, surface area 400–600 m^2^/g) were synthesized using p-type Si wafers with low (10^16^ cm^−3^) and high doping levels (10^19^ cm^−3^), respectively [124,130,131]. This differentiation in synthesis methods is crucial for achieving the desired pore sizes and surface areas necessary for specific applications in hydrogen storage.

**Figure 15:**
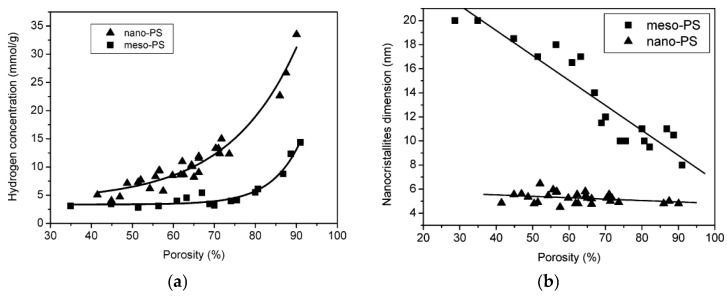
(**a**) Variation in atomic hydrogen concentration along with porosity in meso- (■) and nano-PS (▲) layers. Solid lines added as eye-guides represent exponential fits of the experimental data obtained from FTIR-ATR measurements. (**b**) Dimensions of Si nanocrystallites in meso- (■) and in nano-PS (▲) layers versus porosity estimated by Raman scattering spectroscopy. Straight solid lines represent linear fits of the experimental data [80].

The correlation between the atomic concentration of hydrogen bonded to silicon atoms in PS nanostructures and the porosity of PS layers has been systematically analyzed, as shown in Figure 15a [80]. Meanwhile, Figure 15b illustrates the relationship between porosity and nanocrystallite dimensions, revealing that the size of Si nanocrystallites remains nearly constant across the examined porosity range. This observation suggests that increased porosity is primarily attributed to a reduction in the number of nanocrystallites, rather than significant changes in their individual dimensions.

It was observed that hydrogen concentration values in nano-PS layers exceed those in meso-PS layers throughout the entire porosity spectrum. This constancy in nanocrystallite dimensions implies that the observed increase in hydrogen concentration with porosity can be attributed to a broader hydrogenated region within each nanocrystallite. The thicker hydrogenated layer enhances the material’s ability to store hydrogen efficiently. This finding is critical as it highlights the superior hydrogen storage capacity of nano-PS due to its smaller pore size and higher surface area.

The exponential growth in hydrogen concentration with porosity and the high surface area of nano-PS contribute to its effectiveness in storing large amounts of hydrogen. The nearly constant dimensions of Si nanocrystallites suggest that the rise in porosity is due to a decrease in the number of nanocrystallites. Therefore, the observed increase in hydrogen concentration with porosity can be explained by an overall increase in the thickness of the hydrogenated region within the PS nanocrystallites. By optimizing the synthesis parameters to control the porosity and surface area, researchers can further enhance the hydrogen storage capacity of nano-PS, making it a valuable material for advanced energy storage solutions. These findings provide a strong foundation for developing efficient hydrogen storage materials based on nano-PS, with significant implications for future energy storage technologies.

### 3.3. Hydrogen Storage Effect of Catalyst, Composite Materials, and Porous Silicon as Storage Media

#### 3.3.1. Pd Impregnation in Porous Silicon

The influence of Pd particle impregnation on the behavior of bonded and molecular hydrogen in mesoporous silicon (meso-PS) structures was investigated by Manilov et al. [132] during two processes: (i) PS interaction with water and catalyst, and (ii) molecular H_2_ treatment of PS nanostructures. The study found that higher Pd content in PS correlates with slower hydrogen extraction rates (see Table 1). The presence of Pd particles within the PS layer acts as a physical barrier, preventing the solution from fully penetrating and reacting with the PS. This physical hindrance is a significant factor in reducing the rate of hydrogen extraction. Additionally, an initial silicon oxide layer, which forms a protective barrier, contributes to the reduced hydrogen extraction. This oxide layer further complicates the interaction between the PS and the solution, leading to slower hydrogen evolution.

The study also notes an initial deficiency in SiH_x_ groups, which are essential for hydrogen evolution. The partial oxidation of these groups further reduces the efficiency of hydrogen extraction, as fewer SiH_x_ bonds are available to release hydrogen. The findings from this study have important implications for the design and optimization of hydrogen storage materials. Understanding the dual role of physical and chemical barriers in hydrogen dynamics can guide the development of more efficient hydrogen storage systems. Strategies to mitigate the blocking effects of Pd particles and manage the formation of protective oxide layers will be essential for enhancing the performance of meso-PS as a hydrogen storage material.

#### 3.3.2. Hydrogen Desorption After PS and PS + Pd Treatment in Molecular Hydrogen

The reversible absorption and release of molecular hydrogen by palladium (Pd) metal are key to its high catalytic activity in hydrogenation reactions. The process, described by the equation:

2Pd + xH_2_ → 2PdH_2_; where x ≤ 1, (4)

demonstrates how hydrogen atoms integrate into the Pd lattice, forming palladium hydride. This reversible absorption is fundamental to Pd’s high catalytic activity.

The high catalytic activity of Pd in hydrogenation reactions is attributed to the evolution of atomic hydrogen during the decomposition of Pd hydride. The efficiency of hydrogen absorption and release is directly influenced by the surface area of Pd. Nanodispersed Pd, with its large surface area, is particularly effective, making it a highly desirable material for catalytic applications.

**Figure 16:**
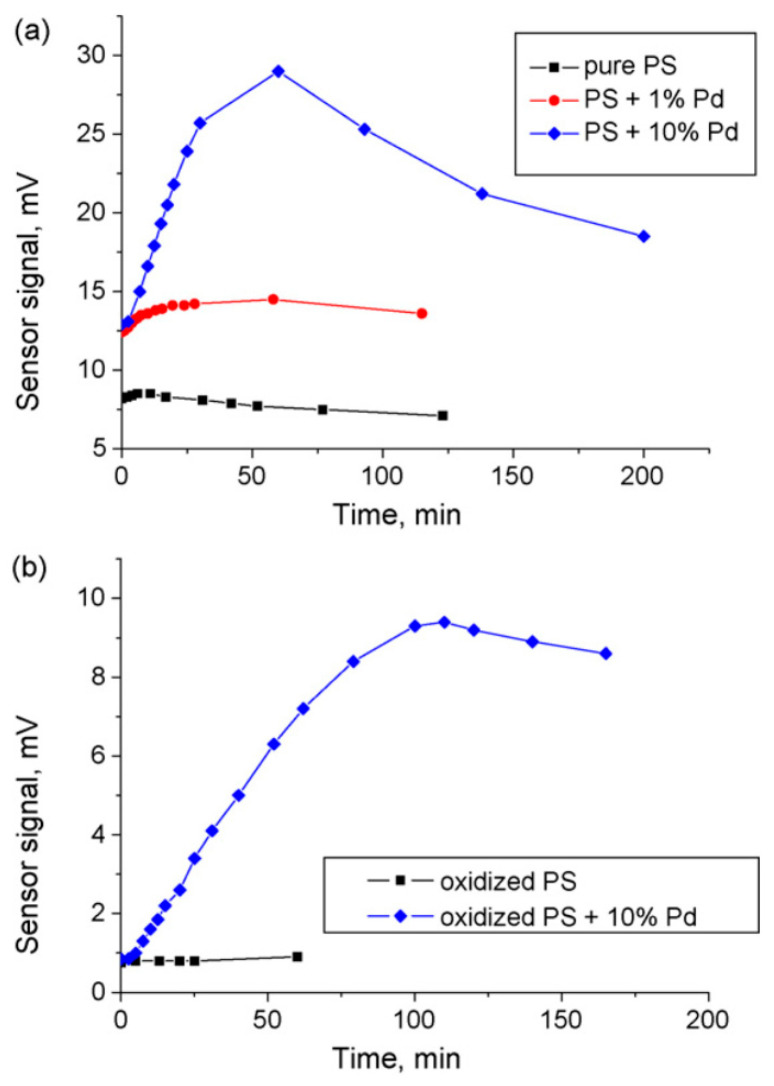
Kinetics of hydrogen extraction from the samples immediately after holding in H_2_: (**a**) initial samples; (**b**) after full PS oxidation [132].

The experiments involving PS + Pd samples provide valuable insights into hydrogen release and desorption behaviors. Figure 16a illustrates the time-dependent hydrogen sensor signal for initial PS and PS + Pd samples following H_2_ treatment [133]. No hydrogen release occurs from PS without Pd. In contrast, PS samples with 1% Pd have increased signal intensity upon placing the sample in the sensor chamber, indicating the release of hydrogen previously absorbed by the Pd particles. However, the sensor was nearing its response limit. In the case of PS samples with 10% Pd, the sensor signal increased linearly during the first 30 min of the experiment, followed by a gradual decay (see Figure 16b). This decay is attributed to the consumption of released hydrogen in the reaction with atmospheric oxygen catalyzed by Pd, potential gas leakage from the hermetic reservoir, and sensor saturation.

#### 3.3.3. Electrochemical Hydrogen Storage in Pd-Coated Porous Silicon/Graphene Oxide

The integration of graphene oxide (GO) and palladium-coated porous silicon (Pd/PS) has been shown to significantly enhance hydrogen storage capacity, demonstrating a synergistic effect between the two materials [133]. A multi-step synthesis process was employed to fabricate the GO-Pd/PS/Si composite, combining the advantages of porous silicon, palladium nanoparticles, and graphene oxide. Initially, Pd nanoparticles were deposited onto PS using an electroless coating method, forming Pd/PS structures. This was followed by spin-coating a graphene oxide sol onto the Pd/PS surface, resulting in the final GO-Pd/PS/Si layered architecture, which effectively optimizes hydrogen adsorption and storage properties.

**Figure 17:**
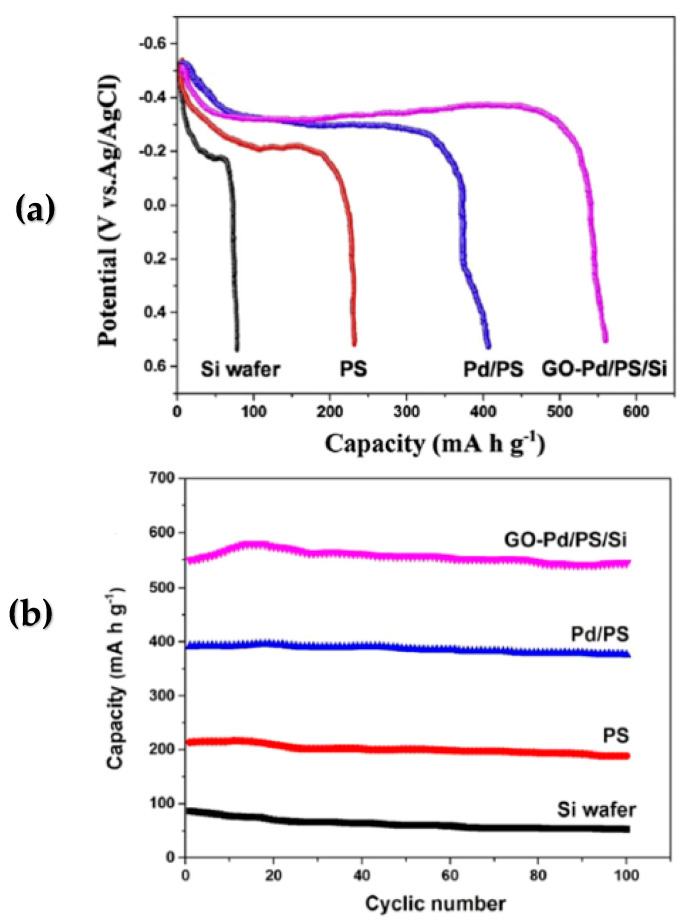
(**a**) Discharge curves of Si wafer, PS, Pd/PS, GO-Pd/PS/Si; (**b**) Cyclic life performance of Si wafer, PS, Pd/PS, and GO-Pd/PS/Si [133].

The electrochemical characterization, including cyclic voltammetry (CV), galvanostatic charge/discharge, and electrochemical impedance spectroscopy (EIS), revealed significant improvements in hydrogen storage capacity. Porous silicon exhibited a higher hydrogen storage capability compared to bulk silicon wafers, with further enhancement observed after Pd nanoparticle coating. The GO-Pd/PS/Si electrode demonstrated the highest hydrogen storage performance, achieving a capacity of 546.1 mAh g^−1^, corresponding to 2.1 wt% hydrogen (see Figure 17a). These findings underscore the synergistic role of Pd and GO coatings in optimizing hydrogen adsorption, charge transfer dynamics, and storage efficiency, making GO-Pd/PS/Si composites a promising platform for advanced hydrogen storage applications.

Furthermore, this electrode exhibited remarkable stability, retaining 99% of its initial capacitance after 100 cycles (Figure 17b). This excellent stability is critical for practical applications, indicating that the material can sustain repeated hydrogen absorption and desorption cycles without significant degradation. The considerable hydrogen storage capacity and stability of the GO-Pd/PS/Si electrode make it a strong candidate for mobile hydrogen storage applications. The combination of high capacity, stability, and the lightweight nature of the materials involved offers significant advantages for portable hydrogen storage solutions.

#### 3.3.4. Effect of Nickel Catalyst on Hydrogen Storage and Desorption in Porous Silicon

Muduli et al.’s research elucidates the synergistic effects of nickel (Ni) as a catalyst on PS, significantly enhancing hydrogen storage capabilities and reducing the thermal desorption energy required [134]. The ball-milling process used to fabricate the Ni-PS composite resulted in a material with a surface area of approximately 146 m^2^/g. This extensive surface area is crucial for maximizing hydrogen storage as it provides numerous unsaturated dangling sites for hydrogen adsorption. The high surface free energy associated with increased porosity further enhances the material’s capacity to store hydrogen.

The study demonstrated that the hydrogen uptake capacity of the composite is highly dependent on the charging pressure and temperature. At 40 bar and 120 °C, the composite exhibited a hydrogen uptake of 1.64 wt%. Increasing the pressure to 60 bar and maintaining a temperature of 60 °C resulted in a higher uptake of 2.69 wt%. These findings indicate that optimizing the pressure and temperature conditions is essential for maximizing hydrogen storage in the composite material. A pressure composition isotherms (PCI) desorption curves demonstrate the presence of approximately 0.5 wt% of trapped hydrogen, which can be mitigated by elevating the desorption temperature. Ni plays a critical role in enhancing hydrogen storage and reducing desorption energy. It functions as an economical catalyst that facilitates the dissociation of molecular hydrogen into atomic hydrogen, which then diffuses into the PS matrix. The presence of Ni nanoparticles on the PS surface increases the number of active sites for hydrogen adsorption, thereby improving the overall hydrogen storage capacity.

Post-hydrogen treatment, the Ni-PS composite undergoes significant structural changes, including the formation of high-energy crystalline phases such as Ni17Si_3_ and NiSi_2_. These phases enhance the material’s crystallinity, which increases from approximately 60% (pre-hydrogenation) to 88%. The catalytic effect of Ni reduces the thermal energy required for hydrogen desorption. The study found that the average decomposition energy requirement decreased, allowing for hydrogen desorption at a lower temperature of 268 °C. This reduction in desorption temperature is beneficial for practical applications, as it lowers the energy cost associated with hydrogen release.

**Figure 18:**
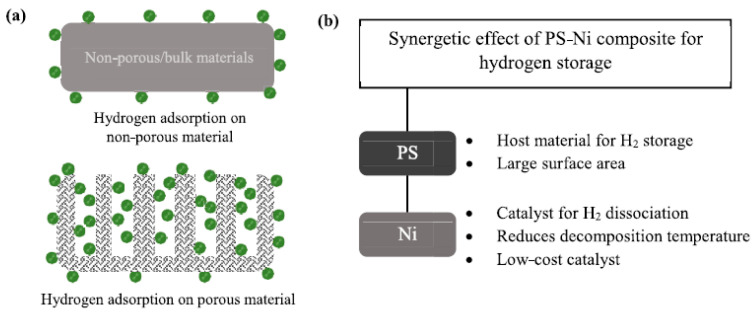
(**a**) Termination of adsorbed hydrogen on non-porous and porous materials (**b**) Advantages of synergetic effect of PS-Ni composite for hydrogen storage [134].

Muduli’s work [134] highlights that increasing porosity in the material leads to a larger surface area, which in turn provides higher surface free energy, making it more suitable for hydrogen storage (Figure 18a). The combined effects of PS and Ni in the composite (Figure 18b) result in three significant outcomes: PS acts as a host material, offering substantial storage capacity; Ni serves as an efficient and cost-effective catalyst, facilitating the dissociation and diffusion of molecular hydrogen within the composite (as described in the equation below); and Nickel also plays a critical role in reducing the temperature required for hydrogen desorption, enhancing the energy efficiency of the process.

Dissociation: H_2_ + Dissociation energy → H + H, (5)

Diffusion: Si–H + Si–Si ↔ Si–H–Si + Dangling bond(6)

**Figure 19:**
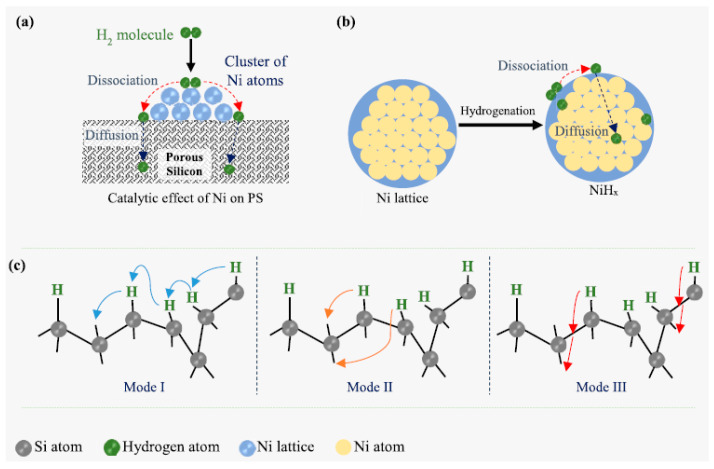
(**a**) Hypothesis of the hydrogen storage mechanism in the PS-Ni composite; (**b**) Enlarged schematic view of hydrogen (**c**) Schematic showing three hydrogen diffusion models in PS [134].

The schematic in Figure 19a (as proposed by Muduli and Kale [134]) depicts the potential hydrogen uptake mechanism: (I) physisorption, where molecular hydrogen attaches to the catalytic surface; Ni nanoparticles bind multiple hydrogen molecules on their surface and dissociate them into atomic hydrogen; (II) some of the dissociated hydrogen atoms infiltrate the Ni lattice, forming NiH_x_ hydrides under high pressure (Figure 19b); and (III) the dissociated hydrogen atoms migrate into the PS through the Ni-Si interface, enhancing the spillover process. Figure 19c illustrates hydrogen diffusion into dangling bonds, detailing three potential modes of hydrogen termination in PS. This detailed mechanism underscores the importance of Ni in enhancing hydrogen storage and facilitating the spillover process.

The synergistic effects of increased surface area, optimized pressure and temperature conditions, and the catalytic activity of Ni result in a highly efficient material for hydrogen storage. These findings contribute valuable insights into the development of advanced materials for energy storage applications.

#### 3.3.5. Enhancing Lithium Hydrides for Hydrogen Storage Using Porous Silicon

The mechanical alloying of LiH with PS is employed to address the challenges posed by the high thermodynamic stability of LiH in hydrogen absorption and desorption [135]. Optimal hydrogen absorption–desorption occurs at 400 °C with a capacity of 3.17 wt%, while the highest uptake, 3.39 wt%, is achieved at 500 °C. Post-milling and heat treatment, the alloy contains multiple Li–Si phases that adjust the thermodynamic stability of the LiH system. The Li–PS alloy undergoes phase transformation (i.e., Li_3_._25_Si ↔ Li_2_._33_Si ↔ Li_4_._4_Si ↔ Si) facilitating reversible hydrogen absorption and desorption, making it highly suitable for applications requiring reversible hydrogen storage. The enthalpies for hydrogen absorption and desorption in the LiH–PS alloy are approximately 94.5 kJ (mol H_2_)^−1^ and 114.9 kJ (mol H_2_)^−1^, respectively, reflecting a significantly lower energy demand compared to most reported LiH alloys. The reduction in hydrogen reaction enthalpy by about 66 kJ (mol H_2_)^−1^ compared to pure LiH highlights the energy efficiency of the alloy. Moreover, the alloying with porous silicon effectively lowers the hydrogen desorption temperature compared to conventional LiH systems, further underscoring its promise as a high-capacity, reversible hydrogen storage material.

To provide a clearer perspective on the performance of Si-based hydrogen storage materials, Table 2 summarizes and compares the key parameters of various systems, including atomic hydrogen content and theoretical mass energy density. The data illustrate how meso- and nano-porous silicon materials perform relative to conventional metal hydrides and other chemical hydrogen storage methods such as hydrolysis and thermolysis. As seen in Table 2, nano- and mesoporous silicon materials demonstrate promising hydrogen storage capacities compared to certain metal hydrides, particularly under ambient conditions. However, chemical hydrides such as NaBH_4_ and NH_3_BH_3_ still exhibit significantly higher hydrogen content and energy density. These comparisons underscore the need for further optimization of Si-based materials, focusing on improving hydrogen uptake and releasing kinetics while maintaining the advantages of safety, reversibility, and cost-effectiveness.

## 4. Applications

### 4.1. Self-Regulated System for Application in Portable Fuel Cells

**Figure 20:**
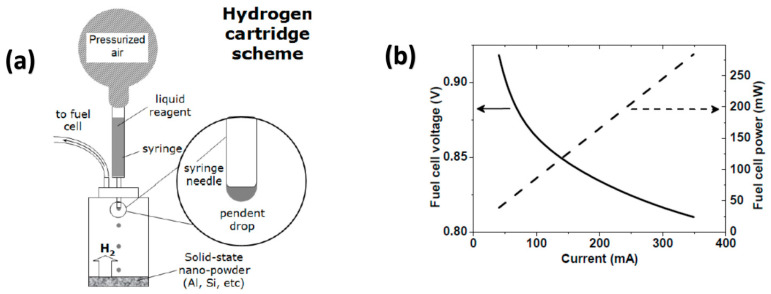
(**a**) Scheme of the double-tank cartridge for hydrogen generation with the use of Al or PS nanopowders. (**b**) Electrical characteristics of the used fuel cell [137].

Pastushenko et al. [137] introduced an innovative method for regulating hydrogen production using aluminum (Al) or PS nanopowders. This novel approach employs a drop-by-drop mechanism within a double-tank hydrogen cartridge eliminating the need for active electrical control due to its self-regulating nature (see Figure 20a). The design ensures that hydrogen production ceases automatically when not required by a fuel cell. The cartridge comprises two tanks connected by a capillary tube. The first tank contains a liquid oxidizer, while the second tank contains PS or Al nanopowder. The self-regulation mechanism operates based on the dynamic pressure difference between the tanks. When this pressure difference exceeds the capillary threshold, the oxidizer flows from the first tank to the second, reacting with the nanopowder to increase hydrogen pressure and reduce the pressure difference. If the pressure difference falls below the capillary threshold, the oxidizer does not flow, and the system remains in standby mode until the hydrogen is consumed by the fuel cell, which reinitiates the cycle by increasing the pressure difference. Numerical analyses were conducted to optimize the cartridge’s parameters, and a prototype was tested using a PaxiTech^®^ fuel cell (Échirolles, France).

The electrical characteristics of the fuel cell, shown in Figure 20b, were measured using a commercial high-purity hydrogen generator and various load resistances. The current and voltage across the load were measured directly, while the fuel cell voltage was calculated, accounting for internal resistance. The results showed a typical decrease in fuel cell voltage with increasing current, especially at low currents below the optimal level of 3A, which yielded maximum power output [138]. The optimal current, which allows for maximum electrical power output for the tested fuel cell, was approximately 3A. In further experiments, the current was kept below 400 mA to maintain optimal performance.

This study presents a significant advancement in hydrogen production technology through a self-regulating hydrogen cartridge system. The drop-by-drop method, devoid of active electrical control, simplifies the regulation process and enhances the system’s efficiency. The prototype demonstrated effective self-regulation and reliable hydrogen production, with promising results when integrated with a fuel cell. The electrical characteristics of the fuel cell indicate its suitability for this application, maintaining efficiency and stability even at varying current loads. This method holds great potential for practical applications in hydrogen fuel cells, offering a cost-effective and efficient solution for hydrogen production and regulation.

### 4.2. Water-Emulsified Diesel Fuel in Diesel Engines

The use of water-emulsified diesel fuel in diesel engines has demonstrated notable benefits, including an extended ignition delay, leading to a higher rate of pre-mixed combustion, increased heat release rate, and higher peak pressures [139]. These emulsions have also been effective in consistently reducing emissions of hydrocarbons (HC), carbon monoxide (CO), carbon dioxide (CO_2_), harmful nitrogen oxides (NO_x_), and particulate matter (PM) [140,141].

In the experimental study conducted by Mehta et al. [142], three types of nanoemulsion fuels were developed, stabilized, and tested in a compression ignition (CI) engine for comparative performance analysis against conventional diesel. These fuels included water-diesel (W/D), nano-aluminum in water-diesel (W/DA), and nano-silicon in diesel with external water injection (W/DS). The study focused on the capability of nano-silicon particles to split water and generate hydrogen, thereby enhancing the CI engine’s efficiency.

The experimental results revealed significant improvements in engine performance when using nanoemulsion fuels. Specifically, the brake specific fuel consumption (BSFC) was reduced by 21% and 37%, while the brake thermal efficiency (BTE) increased by 16% and 14% for W/DA and W/DS, respectively. Additionally, the nanoemulsion fuels demonstrated an increase in induced power. For W/D, the brake mean effective pressure and BTE showed a decrease in NO_x_ emissions due to lower exhaust gas temperatures. However, W/DA and W/DS fuels exhibited a slight increase in NO_x_, CO, HC, and radiative heat emissions, which was attributed to higher peak cylinder pressures and exhaust gas temperatures.

Overall, the study by Mehta et al. demonstrates the potential of nanoemulsion fuels, particularly those incorporating nano-silicon, to improve CI engine performance. Future research should focus on balancing the benefits of enhanced fuel efficiency with the need to control and reduce emissions, ensuring that the advantages of nanoemulsion fuels can be fully realized in practical applications.

### 4.3. Si-Based Agent, a Unique New Antioxidant via Hydrogen

Hydrogen acts as a powerful antioxidant, specifically targeting harmful ROS such as hydroxyl radicals. Antioxidant therapy using hydrogen (often termed hydrogen medicine) has shown promise in alleviating the pathology of various diseases, including brain ischemia–reperfusion (IR) injury, Parkinson’s disease, and hepatitis [143,144], with no reported side effects to date. This positions hydrogen as a highly promising therapeutic agent.

Si-based agents can also be considered as nanodevices that deliver hydrogen, a highly specific antioxidant. Nanoscale Si-based agents have proven to be more effective than those composed of larger particles [145]. This increased effectiveness is due to the interaction between water and the silicon surface, which generates hydrogen, reduces the size of the agent, enhances its surface area, and subsequently produces more hydrogen [146]. As a result, their efficacy has increased over time.

Koyama et al. [60] propose that nano-antioxidant therapy, using Si-based agents, represents an innovative approach that can address the limitations of existing antioxidant therapies (Figure 19). These agents alleviate the symptoms of oxidative stress associated diseases by generating large amounts of antioxidant hydrogen in the body. This approach is also being explored for anti-cancer therapy and holds great potential for improving the treatment of numerous diseases. Moving forward, Si-based agents are expected to contribute to the advancement of nanotherapeutic methods due to their anti-inflammatory, anti-apoptotic, and antioxidant effects.

Si-based agents are reported to have beneficial effects on various organs, including the kidneys, skin, placenta, uterus, and large intestine, where hydrogen generation occurs. Traditional methods of in vivo hydrogen administration, such as inhaling hydrogen gas or drinking hydrogen-rich water, suggest that hydrogen can indirectly impact organs beyond the lungs and digestive tract. Detection of hydrogen in the blood and the possibility that heme protein is a direct target of hydrogen imply that hydrogen’s antioxidant effects are mediated through the bloodstream [147,148]. Studies have confirmed the presence of hydrogen in the blood following the administration of Si-based agents, with blood hydrogen concentration increasing proportionally to the amount of Si-based agent administered. This supports the hypothesis that hydrogen acts on the brain via blood circulation [139,149].

**Figure 21:**
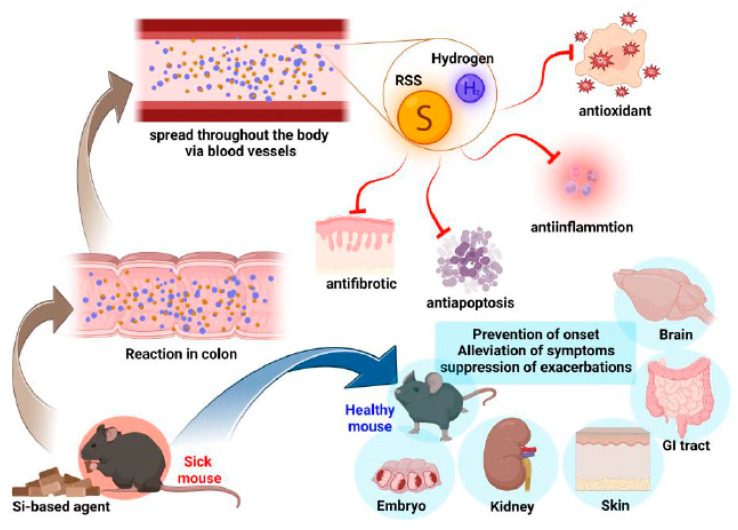
Hypothesis of Si-based agents’ mechanism of action. Si-based agents orally administered to mice via diet react with intestinal juice in the large intestine, resulting in the generation of large amounts of hydrogen and the promotion of RSS production. Hydrogen and RSS are spread throughout the body via the blood. They exert antioxidant, anti-inflammatory, anti-apoptotic, and anti-fibrotic effects on diseased organs (brain, kidneys, intestinal tract, etc.) to improve pathological conditions. RSS: reactive sulfur species. GI: gastrointestinal tract [60].

However, Si-based agents also increase reactive sulfur species (RSS), which are produced in vivo by intestinal bacteria and can enter the bloodstream [150]. This suggests that both hydrogen and RSS can reach other organs via the blood. There are still many issues to be investigated, including the effects of Si-based agents on the intestinal flora and the autonomic nervous system. Nevertheless, considering Koyama’s findings, it is highly likely that the effects of Si-based agents, including their antioxidant properties, are exerted on organs beyond the intestinal tract via the bloodstream (Figure 21).

The ability of Si-based agents to generate hydrogen and RSS, which can travel through the bloodstream, suggests a broader therapeutic impact on multiple organs. Future research should focus on understanding the interactions of Si-based agents with the intestinal flora and the autonomic nervous system to fully harness their potential. The findings support the continued exploration and development of Si-based nanotherapies for comprehensive medical applications.

### 4.4. Si-Based Hydrogen Administration for Ischemia–Reperfusion Injury

Hydrogen is a gas known for its ability to eliminate reactive oxygen species, which play a significant role in ischemia–reperfusion injury (IRI). Since the seminal work by Ohsawa et al. [151], which first demonstrated that H_2_ gas possesses antioxidant properties capable of selectively neutralizing hydroxyl radicals and protecting the brain from IRI, research into the biological and medical applications of H_2_ has expanded rapidly. Several methods for delivering H_2_ have been explored, including inhalation through a ventilator circuit, facemask, or nasal cannula proving effective in reducing IRI across various organs including the brain, heart, lungs, liver, skin, and intestines [147]. However, while inhaled H_2_ gas acts quickly, its practical use in daily clinical settings or for continuous preventive use is limited by the challenges associated with handling high-pressure H_2_ gas. An alternative delivery method is the oral consumption of H_2_-rich water, which is both portable and safe [152]. However, H_2_ has a tendency evaporate over time, potentially leading to its loss before it reaches the stomach or intestines. Injectable H_2_-rich saline offers another delivery option, allowing for more precise control of H_2_ concentrations. This method has shown promise in reducing IRI in the brain (particularly in neonates), heart, lungs, liver, intestines, and kidneys in animal models of oxidative stress [145]. Nonetheless, the amount of H_2_ that can be dissolved in solutions is limited, with water at atmospheric pressure and room temperature able to dissolve only up to 0.8 mM (1.6 mg/L) of H_2_ [153].

Kawamura et al. [145] have introduced an innovative approach to produce significant amounts of H_2_ by reducing silicon to nano-sized particles and allowing these nanoparticles to react with alkaline water. While Si is not an essential nutrient for mammals and its biological role is not fully understood, oral Si supplementation has been reported to be safe for mammals [151]. In their study, Kawamura et al. explored the effectiveness of administering nano-Si in an in vivo model of acute IRI. Their assessments, which included serological, urinary, and histological analyses, revealed that ischemia–reperfusion surgery led to renal function deterioration and tubular epithelial cell damage. However, a diet containing nano-Si significantly mitigated these adverse effects. The protective effect of nano-Si is thought to result from the generation of H_2_ when the nanoparticles react with alkaline water, such as the fluids present in the intestines. Supporting this hypothesis, a diet containing larger Si particles with low H_2_ generation efficiency, did not produce similar protective effects under the same conditions.

Additionally, Kawamura et al. [145] demonstrated that nano-Si treatment provided partial protection to rat kidneys subjected to IRI by reducing oxidative stress through oral administration. This study is the first to show the protective effects of nano-Si in renal IRI. Based on these findings, the potential clinical application of nano-Si for kidney allograft donors prior to transplantation should be considered to improve kidney allograft outcomes.

This method appears promising, as the study demonstrated significant protective effects against renal IRI in animal models. The generation of H_2_ from nano-Si provided substantial protection by reducing oxidative stress, marking the first evidence of such effects in renal IRI.

### 4.5. Application as an Explosive Material

Kovalev et al. [154] have reported on novel heterogeneous hydrogen–oxygen and silicon–oxygen branched chain reactions that demonstrate explosive behavior when pores in hydrogen-terminated PS are filled with condensed or liquid oxygen. This explosive reaction is observed within the temperature range of 4.2–90 K. Infrared vibrational absorption spectroscopy indicates that the Si nanocrystals forming the layers initially have hydrogen-terminated surfaces, with the reaction’s end products being SiO_2_ and H_2_O. Time-resolved optical experiments reveal that the explosive reaction occurs within an exceptionally brief period of 10^−6^ s. The study highlights the significant role of the unique structural properties of porous Si layers in enabling this intense explosive interaction. These insights could have significant implications for understanding the behavior of silicon-based materials in various applications, particularly those involving cryogenic environments and reactive gases.

## 5. Conclusions and Perspectives

Hydrogen is widely recognized as a promising clean energy carrier due to its high energy density and zero carbon emissions during use. However, the widespread adoption of hydrogen energy depends on developing new and efficient technologies for both its generation and storage. Solid-state materials, including silicon-based nanostructures, offer an attractive approach because hydrogen can be stored and released through chemical and physical adsorption processes under relatively mild conditions.

Research summarized in this review shows that Si nanoparticles, porous Si, and Si nanowires interact with water or alcohol solutions to release hydrogen through oxidation and hydrolysis reactions, while porous Si nanostructures with high internal surface areas covered by SiHx groups enable controlled hydrogen release via thermal decomposition or chemical reactions with water/alkali. These properties, combined with the ability to tune porosity, morphology, and surface chemistry, position Si-based nanomaterials as promising candidates for solid-state hydrogen storage systems.

At the same time, DOE guidelines emphasize that practical hydrogen storage materials should achieve gravimetric capacities above 6.5 wt% and enable hydrogen release at −20 to +100 °C under ambient pressure. While current Si-based materials do not yet meet these strict targets, they offer unique advantages for niche applications. In particular, portable power sources for drones [155], robotics, and mobile devices could benefit from safe, small-scale, and precisely controlled hydrogen release systems, where high-pressure tanks or cryogenic methods are impractical.

Recent advances also highlight that structural and surface engineering of Si-based materials can substantially enhance both hydrogen generation efficiency and overall energy performance. Studies on functionalized microporous Si architectures, Zintl-phase alloys, and interface-modified micro-Si systems collectively demonstrate that rational material design can overcome kinetic and stability barriers, opening new opportunities for energy storage and conversion applications [156,157,158].

Another emerging direction is the biomedical field. Hydrogen has shown therapeutic potential as a selective antioxidant against harmful reactive oxygen species, and Si-based nanomaterials, being bioinert and capable of generating hydrogen under mild physiological conditions, could enable hydrogen-based medical therapies that other storage methods cannot support.

Looking forward, research should focus on enhancing storage capacity, improving reversibility and cyclability, and optimizing hydrogen release kinetics under mild conditions. Moreover, integrating Si-based materials into practical device architectures—both for energy and medical applications—will require multidisciplinary collaboration across materials science, catalysis, thermodynamics, and biomedical engineering.

Overall, while Si-based hydrogen storage cannot compete with compressed or liquid hydrogen for large-scale energy applications, it offers significant potential for portable power systems and hydrogen medicine, aligning with future needs for sustainable, safe, and innovative energy and medical technologies.

## Figures and Tables

**Table 1:** Following chemical treatment of the studied samples for 18 h at room temperature, total amounts of H_2_ were extracted [132].

Sample	Hydrogen Volume, mL/g × 10^−3^
PS	1.2–1.3
PS + 1%Pd	0.90–1.14
PS + 1%Pd with H_2_ treatment	1.04–1.27
PS + 10%Pd	0.52–0.65
PS + 10%Pd with H_2_ treatment	0.17–0.65

**Table 2:** Comparison of hydrogen content and theoretical energy density for Si-based materials and other representative hydrogen storage systems.

Materials	Atomic Hydrogen Content, (mmol g^−1^)	Theoretical Mass Energy Density, (W-h kg^−1^)	Ref.
meso-PS (90%, 10 nm)	13	429	[80]
nano-PS (90%, 5 nm)	34	1120	[80]
nano-PS powder (>95%, 2–3 nm)	66	2176	[80]
reversible metal hydrides			[136]
MgH_2_ →Mg + H_2_	76	2505	
LaNi_5_H_6_ → LaNi_5_ + 3H_2_	14	461	
hydride hydrolysis			[136]
(NaBH_4_ + 2H_2_O) →NaBO_2_ + 4H_2_	108	3560	
(LiBH_4_ + 4H_2_O) → LiOH + H_3_ BO_3_ + 4H_2_	85	2802	
hydride thermolysis			[136]
NH_4_BH_4_ → BN + 4H_2_	244	8043	
NH_3_BH_3_ → BN + 3H_2_	195	6428	

## Data Availability

Data sharing is not applicable. No new data were created or analyzed in this study.
